# M1-primed human corneal stromal stem cell-derived exosomes delivering protein-coding transforming growth factor-β3 RNA promote corneal regeneration

**DOI:** 10.1093/stcltm/szag079

**Published:** 2026-09-04

**Authors:** Mithun Santra, Elizabeth Rubin, Syeda R Ali, Moira L Geary, Christine Chandran, Julia T Coelho, Ella J Dewald, Xi Cheng, Martha L Funderburgh, Deepinder K Dhaliwal, Xin D Gao, Vishal Jhanji, Gary H F Yam

**Affiliations:** Department of Ophthalmology, Corneal Regeneration Lab, University of Pittsburgh School of Medicine, Pittsburgh, PA 15219, United States; Department of Ophthalmology, Corneal Regeneration Lab, University of Pittsburgh School of Medicine, Pittsburgh, PA 15219, United States; Department of Ophthalmology, Corneal Regeneration Lab, University of Pittsburgh School of Medicine, Pittsburgh, PA 15219, United States; Department of Ophthalmology, Corneal Regeneration Lab, University of Pittsburgh School of Medicine, Pittsburgh, PA 15219, United States; Department of Ophthalmology, Corneal Regeneration Lab, University of Pittsburgh School of Medicine, Pittsburgh, PA 15219, United States; Department of Ophthalmology, Corneal Regeneration Lab, University of Pittsburgh School of Medicine, Pittsburgh, PA 15219, United States; Department of Ophthalmology, Corneal Regeneration Lab, University of Pittsburgh School of Medicine, Pittsburgh, PA 15219, United States; Department of Ophthalmology, University of Pittsburgh School of Medicine, Pittsburgh, PA 15219, United States; Department of Ophthalmology, Corneal Regeneration Lab, University of Pittsburgh School of Medicine, Pittsburgh, PA 15219, United States; Department of Ophthalmology, Corneal Regeneration Lab, University of Pittsburgh School of Medicine, Pittsburgh, PA 15219, United States; Department of Ophthalmology, University of Pittsburgh School of Medicine, Pittsburgh, PA 15219, United States; Department of Bioengineering, University of Pittsburgh, Pittsburgh, PA 15219, United States; Institute for Synthetic Biology, University of Pittsburgh, Pittsburgh, PA 15219, United States; Department of Ophthalmology, Corneal Regeneration Lab, University of Pittsburgh School of Medicine, Pittsburgh, PA 15219, United States; Department of Ophthalmology, Corneal Regeneration Lab, University of Pittsburgh School of Medicine, Pittsburgh, PA 15219, United States; McGowan Institute for Regenerative Medicine, University of Pittsburgh, Pittsburgh, PA 15219, United States

**Keywords:** corneal stromal stem cells, exosomes, transforming growth factor β3, protein-coding, corneal opacity

## Abstract

**Introduction:**

Human corneal stromal stem cells (hCSSCs), which exhibit mesenchymal stem cell (MSC) features, prevent fibrotic scarring and promote regeneration of injured stromal tissue, thereby restoring corneal clarity in mice. These therapeutic effects rely on hCSSCs’ ability to respond to early inflammatory cues, suppress chronic neutrophil infiltration, and inhibit fibrosis. In this study, we examined the responses of hCSSCs to transient pro-inflammatory M1 stimulation and characterized the exosomes (Exo) they secreted.

**Methods:**

We evaluated the expression of full-length protein-coding human *TGFB3* mRNA molecules in purified Exo, and investigated Exo uptake, induction of human transforming growth factor β3 (hTGFβ3) protein expression, and the effects of different hTGFβ3/β1 ratios on myofibroblast generation from human corneal stromal keratocytes (CSKs) and stromal fibroblasts (SFs). The anti-scarring effect of hCSSC-Exo enriched in *TGFB3* mRNA was assessed in a mouse model of acute corneal stromal injury.

**Results:**

Our results showed that hCSSC-Exo contained *TGFB3* mRNA transcripts, and their abundance increased following pro-inflammatory M1 stimulation. Exo uptake enhanced hTGFβ3 protein expression in hCSKs, and a higher hTGFβ3/β1 ratio in hCSKs and hSFs suppressed expression of α-smooth muscle actin and TGFβ1-induced myofibroblast differentiation. In vivo, topical treatment with M1-primed hCSSC-Exo reduced corneal opacity compared with sham-treated controls. Notably, hCSSC-Exo enriched with *TGFB3* mRNA demonstrated greater scar-reducing and wound-healing efficacy.

**Conclusions:**

M1-primed hCSSC-Exo containing protein-coding *TGFB3* mRNA significantly inhibited corneal opacity and promoted scar-free healing in injured mouse corneas. These findings support the anti-fibrotic and regenerative potential of hCSSC-Exo, which can be developed as a cell-free therapeutic strategy for corneal fibrosis and scarring.

## Graphical Abstract

**Graphical Abstract:**
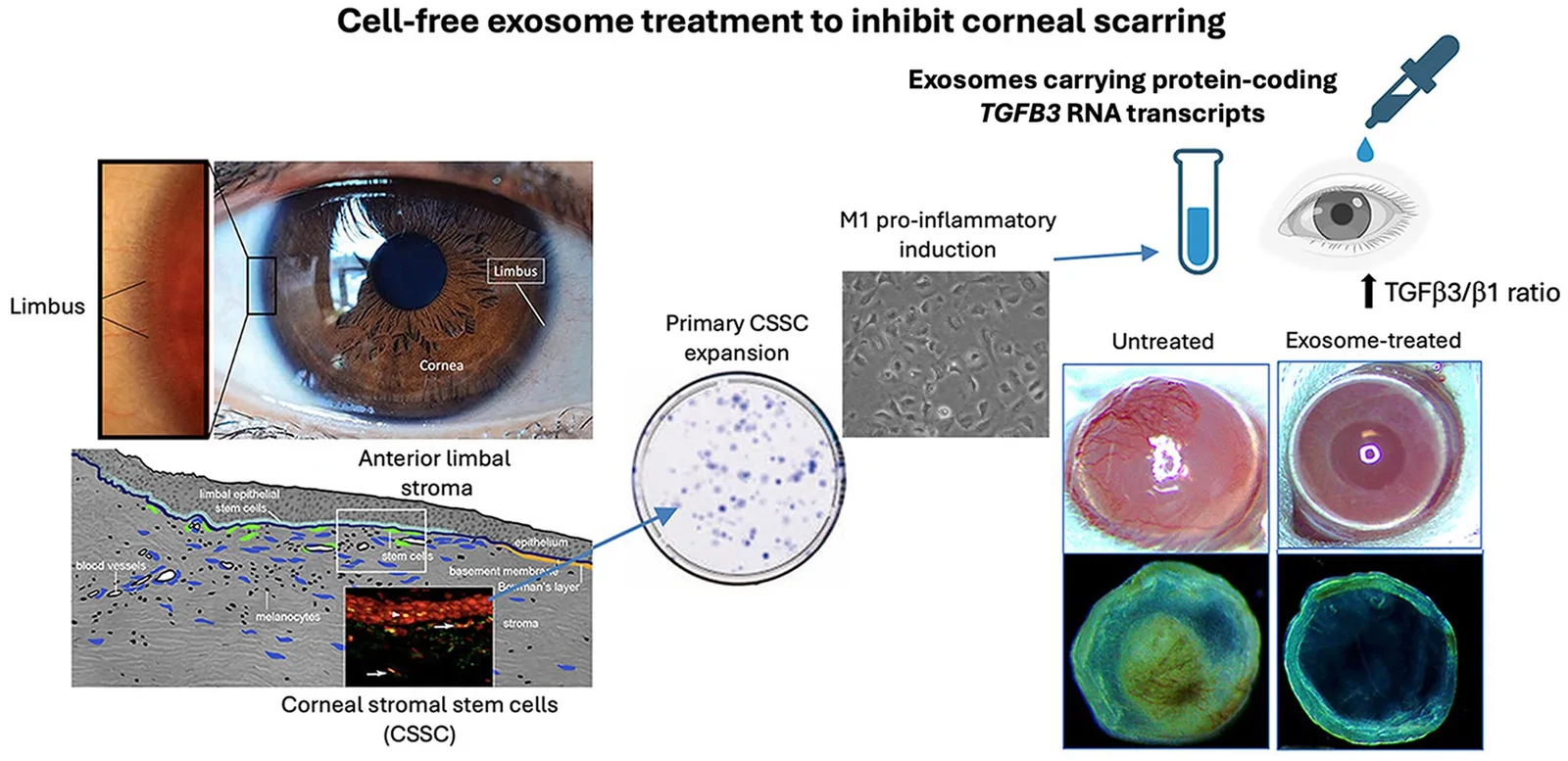

Significance statement Human corneal stromal stem cells (hCSSCs) secrete exosomes containing protein-coding transforming growth factor β3 (*TGFB3*) mRNA, and its expression is upregulated under M1 pro-inflammatory conditions. Following uptake by corneal stromal cells, these exosomes increase the cellular TGFβ3/β1 ratio and suppress myofibroblast differentiation. In a mouse model of corneal stromal injury, topical administration of *TGFB3* mRNA-enriched hCSSC-derived exosomes significantly reduced corneal scarring and opacity. These findings present a novel cell-free therapeutic approach for preventing corneal fibrosis and restoring corneal transparency.

## Introduction

A transparent cornea is essential for normal vision, as it transmits and refracts light onto the retina. However, in everyday life, the cornea can develop haze, clouding, and opacities resulting from trauma, infections, and chemical or thermal burns, all of which can compromise its structure and function. When injured, the cornea exhibits inflammatory and fibrotic responses. Pro-fibrotic cytokines induce the transition of native corneal stromal keratocytes (CSKs) into repair-type stromal fibroblasts (SFs) and α-smooth muscle actin (αSMA)-expressing myofibroblasts (myoFs).^1^^,^^2^ These cells lose their native keratocyte features and deposit excessive, disorganized extracellular matrix (ECM), resulting in permanent corneal scarring and opacity.^3^^,^^4^ Corneal opacity is a major cause of blindness worldwide, affecting more than 12 million people (Eye Bank Association of America; https://restoresight.org/cornea-donation/). Although corneal transplantation can restore vision, its success is limited by immune rejection, the need for prolonged immunosuppression, and, most importantly, the global shortage of donor corneas.^5^ Hence, developing regenerating treatments that suppress fibrosis and restore corneal transparency without relying on donor tissue represents a critical unmet clinical need.

Preclinical studies have shown that human corneal stromal stem cells (hCSSCs), the progenitors of CSKs, suppress corneal fibrosis after injury, evidenced by downregulation of fibrosis-associated markers, including collagen type III (COL3), fibronectin (FN), tenascin C (TNC), and αSMA, while restoring the highly organized stromal collagen architecture.^6–10^ Our group identified transforming growth factor β3 (TGFβ3) as a key mediator of this anti-fibrotic effect.^11^ In contrast to pro-fibrotic TGFβ1, TGFβ3 promotes regenerative healing, and an increased TGFβ3/β1 expression ratio favors scar-reducing or scar-free tissue repair.^12^^,^^13^ Small interfering RNA (siRNA)-mediated *TGFB3* knockdown significantly diminished the anti-scarring activity of hCSSCs.^11^ Other studies also demonstrate that TGFβ3 preserves a non-fibrotic ECM and normal corneal stromal architecture.^14^^,^^15^

Transient stress responses enhance stem cells’ therapeutic potential by promoting survival, maintaining homeostasis, and improving regenerative function.^16^^,^^17^ These adaptive actions often involve intrinsic signaling, such as heat shock response and trophic factors.^18^ The exposure to inflammatory milieu boosts paracrine signaling via production of anti-inflammatory mediators such as TGFβ3, indoleamine 2,3-dioxygenase, and prostaglandin E2.^19^^,^^20^ Other transient stressors include mechanical, nutrient-deprived, hypoxic, and metabolic conditions.^21^ In CSSCs, coculture with M1-polarized macrophages similarly enhances immunomodulatory and regenerative capacity through increased TGFβ3 expression and reduced production of inflammatory cytokines.^11^^,^^22^

This study investigates the paracrine signaling of hCSSCs pre-conditioned by transient stress to stimulate anti-scarring activity and explores the potential of cell-free treatment to overcome the limitations of corneal transplantation. We hypothesize that hCSSC-derived exosomes (hCSSC-Exo) carry protein-coding *TGFB3* mRNA, which is enriched following non-lethal stress preconditioning of parental cells. Our previous studies have shown that hCSSC-Exo deliver anti-fibrotic microRNAs, including miR-29a and 381,^23^ and that treatment with hCSSCs or Exo significantly reduces corneal opacity and fibrosis in mouse models.^7^^,^^9^^,^^10^^,^^24^^,^^25^ In addition, compressed collagen gels encapsulating hCSSCs inhibited scarring in rabbit corneas,^26^ highlighting a non-contact therapeutic mechanism. Here, we propose that hCSSC-Exo-mediated delivery of protein-coding *TGFB3* mRNA induces an amplifiable and sustained TGFβ3 production in recipient stromal cells, thereby suppressing fibrosis and promoting corneal regeneration.

## Materials and methods

Materials and methods, and statistical analyses in Supplementary Information.

## Results

### Human CSSCs possessed unique phenotypes different from CSKs and SFs

**Figure 1.**
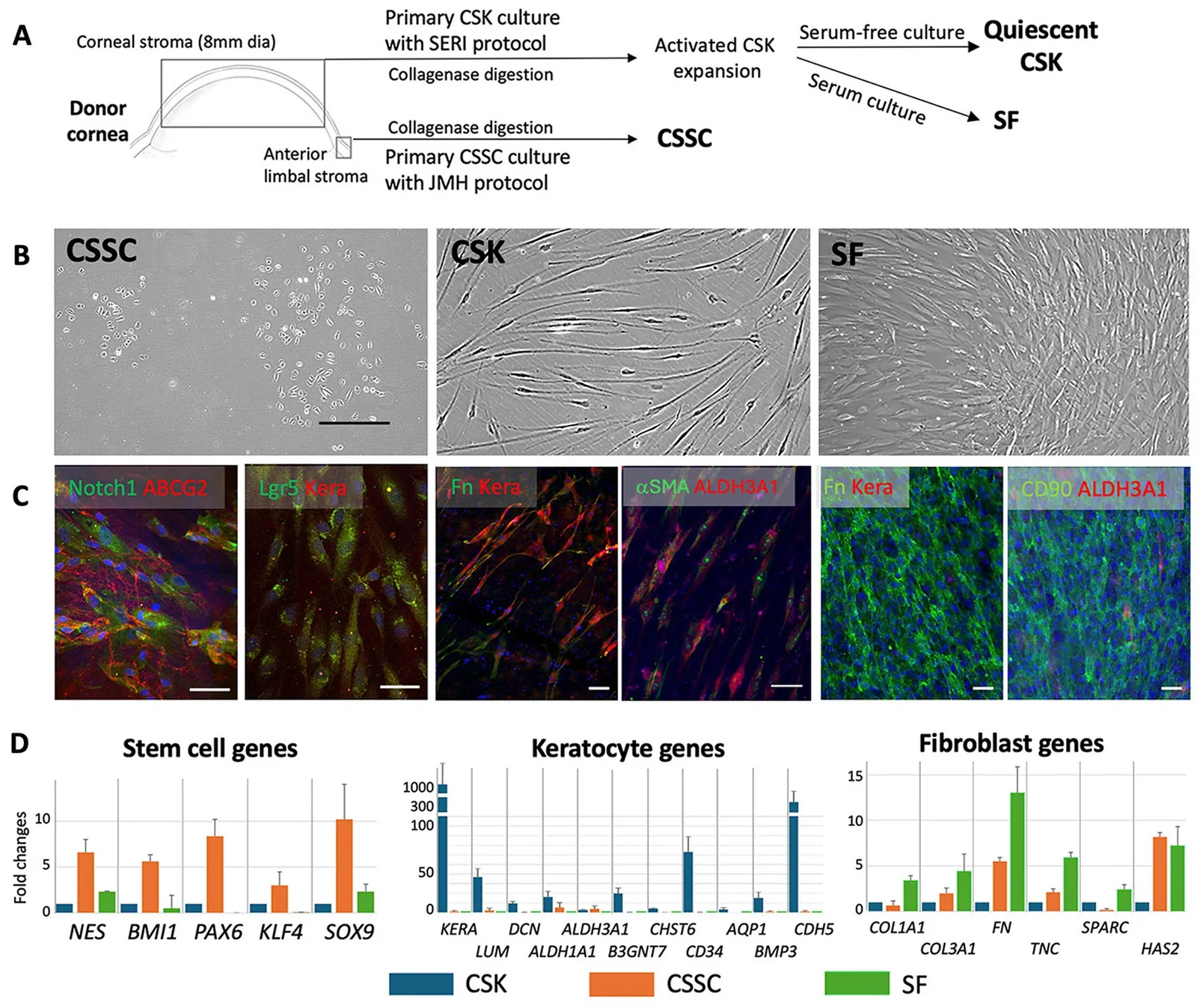
Corneal stromal cell isolation, culture, and characterization. (A) A schematic diagram illustrates the tissue sources of CSSCs, CSKs, and SFs. Central stroma from a healthy donor cornea was harvested for CSK isolation and culture using the SERI protocol with 0.5% serum. Activated CSKs were transitioned to serum-free conditions to induce quiescence. SFs were generated by culturing CSKs in the presence of 5% serum for at least two passages. CSSCs were isolated from the anterior limbal stroma and cultured using a reported JM-H protocol. (B) Phase contrast images displayed primary human CSSCs, CSKs, and SFs. Scale bar: 100 µm. (C) Immunofluorescence shows expression of phenotypic markers for hCSSCs: Notch1, ABCG2, Lgr5; CSKs: keratocan (Kera), aldehyde dehydrogenase 3A1 (ALDH3A1); and SFs: fibronectin (Fn), CD90, and negligible αSMA. Scale bars: 20 µm. (D) Gene expression profiles of stem cell, keratocyte, and fibroblast markers in primary hCSSC, hCSK, and hSF cultures. Data are represented as mean ± SD from triplicate experiment.

Three donor corneas, HC579, HC584, and HC670 (Supplementary Table S1), were processed to cultivate hCSSCs and hCSKs. SFs were generated from serum-added cultures of hCSKs (Figure 1A). Primary hCSSCs exhibited clonal growth and expressed ATP-binding cassette transporter glycoprotein 2 (*ABCG2*), *NOTCH1*, Leucine-rich repeat-containing G-protein coupled receptor 5 (*LGR5*), Nestin (*NES*), B lymphoma Mo-MLV insertion region1 (*BMI1*), Paired box 6 (*PAX6*), Krüppel-like factor 4 (*KLF4*), and SRY-box transcription factor 9 (*SOX9*), at higher levels than CSKs and SFs (Figure 1B and C; orange bars in Figure 1D), similar to previous reports.^6^^,^^27^ In contrast, quiescent hCSKs expressed keratocyte-specific genes, including keratocan (*KERA*), lumican (*LUM*), decorin (*DCN*), aldehyde dehydrogenase *ALDH1A1* and *ALDH3A1*, *B3GNT7* (UDP-GlcNAc-βGal β1-3, N-acetylglucosaminyl-transferase 7), *CHST6* (carbohydrate sulfotransferase 6), *CD34*, aquaporin-1 (*AQP1*), and cadherin 5 (*CDH5*), whereas *ACTA2* (encoding αSMA) was negligibly detectable (Figure 1B and C; blue bars in Figure 1D).^28–31^ hSFs, in contrast, showed extensive growth and negligibly expressed *KERA* and *ALDH3A1* but were positive for *CD90* and fibrosis-associated genes—*COL1A1*, *COL3A1*, *FN*, *TNC*, Secreted Protein Acidic and Cysteine Rich (*SPARC)*, and hyaluronan synthase-2 (*HAS2*) (Figure 1B and C; green bars in Figure 1D).

### Pro-inflammatory M1 stress upregulated TGFB3 mRNA expression in hCSSCs

**Figure 2.**
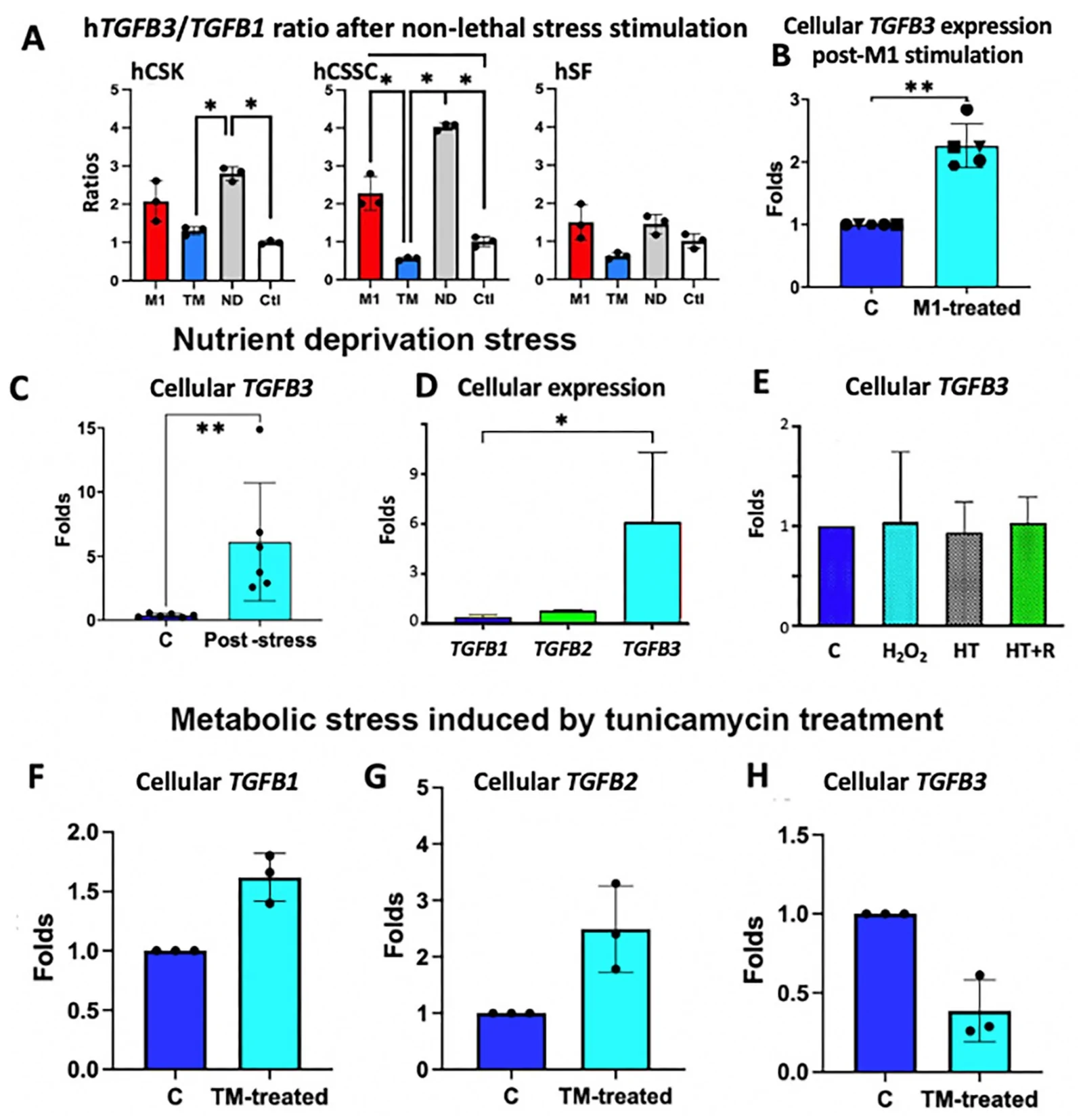
Transient non-lethal stress modulated TGFβ expression in hCSSCs. (A) Donor CSSCs displayed increased h*TGFB3*/*TGFΒ1* ratios in response to pro-inflammatory M1 and nutrient deprivation (ND) conditions, while CSKs and SFs exhibited minor or negligible changes. (B) Multiple CSSC batches from different donor corneas (HC436, HC439, HC515, HC540, HC624, and HC641) consistently demonstrated *TGFB3* upregulation following co-culture with M1-RAW conditioned media. (C, D) Nutrient deprivation (serum-free) stress triggered *TGFB3* upregulation in multiple CSSC batches, with no significant changes in *TGFB1* and *TGFB2* expression. (E) *TGFB3* expression remained unchanged under other transient stresses, including H_2_O_2_ oxidative and heat-shock (HT) stress with and without isothermal recovery (R). (F-H) Metabolic ER stress caused by tunicamycin (TM) treatment increased *TGFB1* and *TGFB2* expression but downregulated *TGFB3*. Data are represented as mean ± SD from triplicate experiment. *P < .05; **P < .01, Mann-Whitney *U*-test.

We assessed TGFβ expression changes in corneal stromal cells following exposure to non-lethal stressors, including pro-inflammatory, metabolic, oxidative, heat-shock, and nutrient-deprived (ND, serum-free) conditions (Supplementary Figure S5; methods in Supplementary Information). Donor-matched hCSK, hCSSC, and hSF cultures were treated with conditioned media (CM, at 100 µg protein/mL) from M1 mouse macrophages (RAW264.7), or ND for 48 h. Cell lysates were analyzed for *TGFB* gene expression, and the expression ratios of *TGFB3/B1* were compared. hCSSCs showed significantly higher *TGFB3/B1* ratios after M1-RAW-CM treatment compared to naive control (*P *< .05, Mann-Whitney *U* test) (Figure 2A). M1-induced *TGFB3* upregulation was consistently observed in hCSSCs from different donor corneas (HC436, HC439, HC515, HC540, HC624, and HC641) with a mean increase of 2.1 ± 0.1 fold over controls (*P *= .007, Mann-Whitney *U* test) (Figure 2B). Similar but insignificant changes were observed in hCSKs, where M1 stress moderately upregulated *TGFB3/B1* ratios (Figure 2A). In contrast, hSFs did not show any changes. These results indicate that *TGFB3* is upregulated in hCSSCs in response to pro-inflammatory M1 condition. Under ND culture, *TGFB3* was significantly upregulated in hCSSCs by 6.1 ± 4.2 fold relative to untreated control (*P *= .002, Mann-Whitney *U* test) (Figure 2C), resulting in higher *TGFB3/B1* ratios, a pattern also seen in hCSKs but not in hSFs (Figure 2A). The relatively large standard deviation likely reflects donor-to-donor variation in baseline *TGFB3* expression among hCSSC batches. In contrast, neither *TGFB1* nor *TGFB2* expression was affected (Figure 2D).

Other transient stressors did not enhance *TGFB3* expression in hCSSCs. Metabolic ER stress induced by tunicamycin (TM) (2.5 µg/mL for 48 h) activated unfolded protein response, evidenced by *XBP1* (X-box binding protein-1) mRNA splicing (Supplementary Figure S6) and upregulation of ER stress- and apoptosis-related genes (*BIP/GRP78*, *CHOP*, *PERK*, *ATF4* and *ATF6*) (Supplementary Figure S7). Under these conditions, *TGFB3* expression reduced by 61 ± 16% (Figure 2H), whereas *TGFB1* and *TGFB2* increased by 1.6 ± 0.2 fold and 2.5 ± 0.6 fold, respectively, compared with untreated controls (Figure 2F and G), suggesting a shift toward pro-fibrotic phenotype. In contrast, oxidative stress caused by H_2_O_2_ and hyperthermia (HT), with or without isothermal recovery (HT + R), had no significant effect on *TGFB3* expression in hCSSCs (Figure 2E).

### hCSSC-derived exosomes contained protein-coding TGFB3 RNA

Exo from 13 donor-derived CSSC batches were purified (Table S4). As reported, they exhibited characteristic spherical morphology under transmission electron microscopy, with diameter ranging from 60 to 135 nm as determined by tunable resistive pulse sensing using the qNano Gold System (Supplementary Figure S8).^23^ Flow cytometry and western blot analyses confirmed expression of Exo markers CD9, CD63, and CD81 (Supplementary Figure S9).

**Figure 3.**
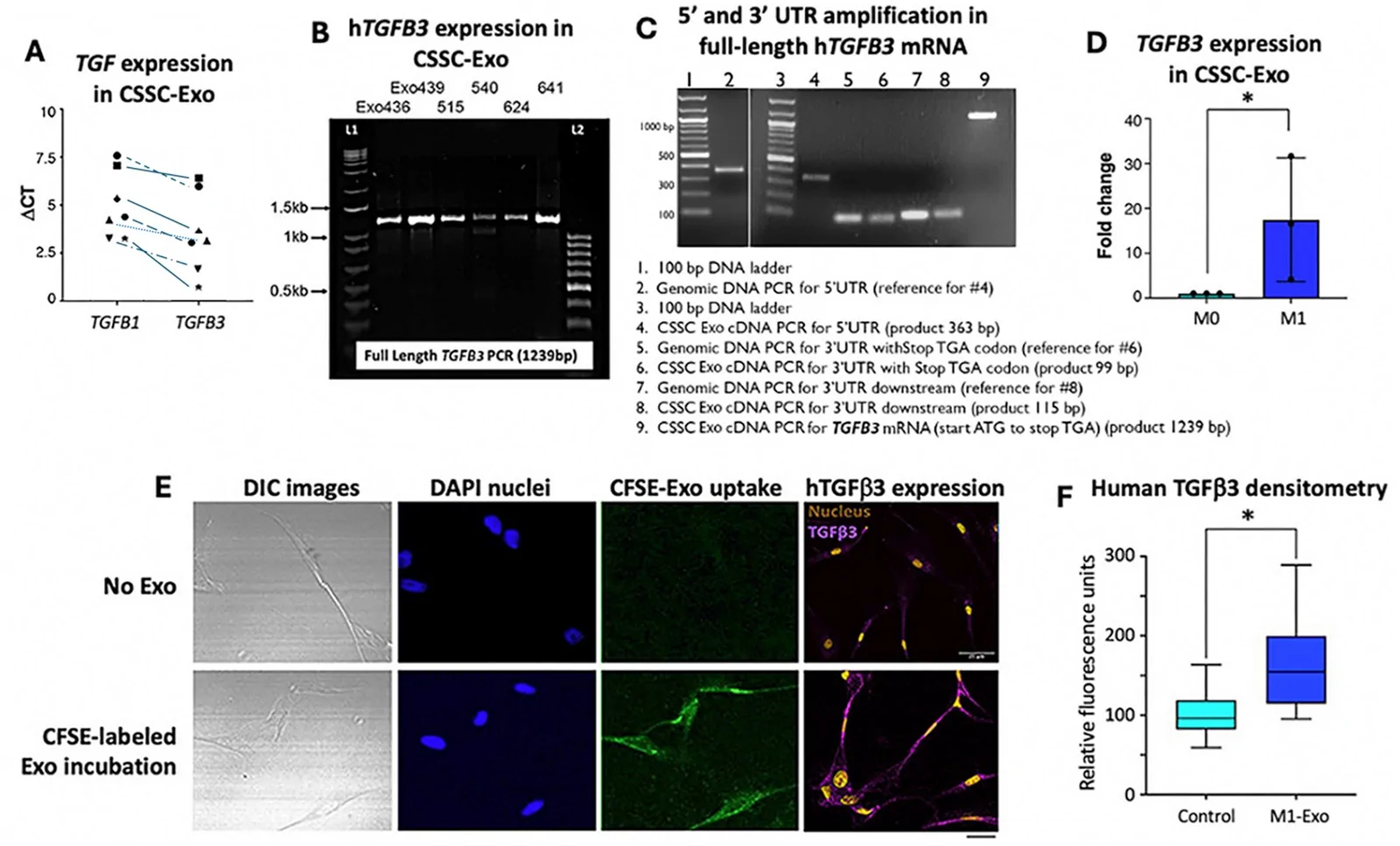
M1 pro-inflammatory treatment modulated TGFβ expression in hCSSCs and exosomes (Exo). (A) Six donor CSSC batches (HC436, 439, 515, 540, 624, and 641) were treated with M1 RAW-CM (100 µg protein/mL). Purified Exo from all six batches consistently exhibited lower *U6*-normalized delta CT (ΔCT) values for *TGFB3* than for *TGFB1*, indicating higher relative *TGFB3* expression in paired comparison. (B) Primers specifically designed to amplify full-length human *TGFΒ3* mRNA (see Supplementary Figure S2) generated a single PCR product of 1239 bp from Exo samples across different donor CSSCs, as shown by agarose gel electrophoresis. (C) Gel image showing PCR products for the 5’UTR (363 bp) (lane 4), stop codon-containing 3’UTR (99 bp) (lane 6), and downstream 3’UTR (115 bp) (lane 8), with the corresponding reference control using genomic DNA (lanes 2, 6, and 7). Lane 9 indicates FL hTGFB3 mRNA from start ATG to stop TGA codon. (D) QPCR analysis revealed significant upregulation of *TGFB3* RNA (normalized to housekeeping *U6*) in M1-stimulated hCSSC-Exo relative to M0 RAW-CM-treated samples (HC436, HC515, and HC641) (*P < .05; Mann-Whitney *U* test). (E) Uptake of CFSE-labeled hCSSC-Exo by hCSKs induced human TGFβ3 protein expression, whereas negligible hTGFβ3 expression was observed in cells without Exo treatment. Scale bar: 25 µm. (F) Signal densitometry demonstrates significant upregulation of hTGFβ3 protein in hCSKs (*n* > 50 cells) that internalized CSSC-Exo containing *TGFB3* RNA transcripts (*P < .05; paired Student’s *t*-test).

Following normalization to housekeeping RNA *U6*, exosomal *TGFB3* mRNA was consistently more abundant than *TGFB1* mRNA, as indicated by lower ΔCT values in six hCSSC batches (Figure 3A).

To determine whether exosomal *TGFB3* mRNA transcripts were intact full-length molecules, including 5′ untranslated region (UTR), protein-coding sequence, and 3’UTR, conventional PCR was performed using cDNA synthesized with oligo(dT) primers that anneal to the poly(A) tail and specific primer sets described in  Supplementary Figure S2. Agarose gel electrophoresis revealed a single PCR product of 1239 bp, corresponding to the complete coding sequence spanning the translation start codon (ATG; nt1086, NM_001329939.2) to the stop codon (TGA; nt2325) (Figure 3B, band 9). PCR products with expected size were detected for the 5’UTR (363 bp), stop codon-containing 3’UTR (99 bp), and downstream 3’UTR (115 bp) (Figure 3C, bands 4, 6, 8). Comparable band intensities of the 5’UTR and downstream 3’UTR amplicons (band 4 compared with band 8, intensity ratio ∼ 1) indicate intact full-length *TGFB3* mRNA molecules delivered in hCSSC-Exo cargo.

### Upregulation of TGFB3 RNA in exosomes after CSSCs exposed to M1 pro-inflammatory stimulus

Following treatment with M1 RAW-CM, hCSSC-Exo from HC436, HC515, and HC641 showed significantly higher levels of *TGFB3* mRNA, normalized to *U6*, than their respective M0 RAW-CM-treated controls (*P *< .05; Mann-Whitney *U*-test; Figure 3D). *TGFB3* mRNA abundance was increased by 31.6-fold in HC436-Exo, 16.6-fold in HC515-Exo, and 4.2-fold in HC641-Exo. These results indicate that Exo cargo content reflects molecular changes in their parental cells and that pro-inflammatory stimulation enhances the packaging of *TGFB3* mRNAs into Exo.

### Uptake of M1-stimulated CSSC-Exo induced hTGFβ3 protein expression in hCSKs

hCSKs were incubated with CFSE-labelled hCSSC-Exo (method in Supplementary Information). Within 4 h, Exo were internalized, as demonstrated by CFSE fluorescence localized to the perinuclear cytoplasm and along cell processes (Supplementary Figure S10). Production of hTGFβ3 protein was assessed by immunofluorescence. Distinct cytoplasmic TGFβ3 immunoreactivity was detected 24 h after treatment with hCSSC-Exo (Figure 3D). Quantitative fluorescence densitometry showed a significant increase in TGFβ3 protein expression compared with untreated cells (*n* > 50 cells) (Figure 3E). These findings indicate an efficient uptake of hCSSC-Exo by recipient CSKs and translation of the delivered mRNA into TGFβ3 protein.

### M1-primed CSSC-Exo induced anti-fibrotic activity in vitro

**Figure 4.**
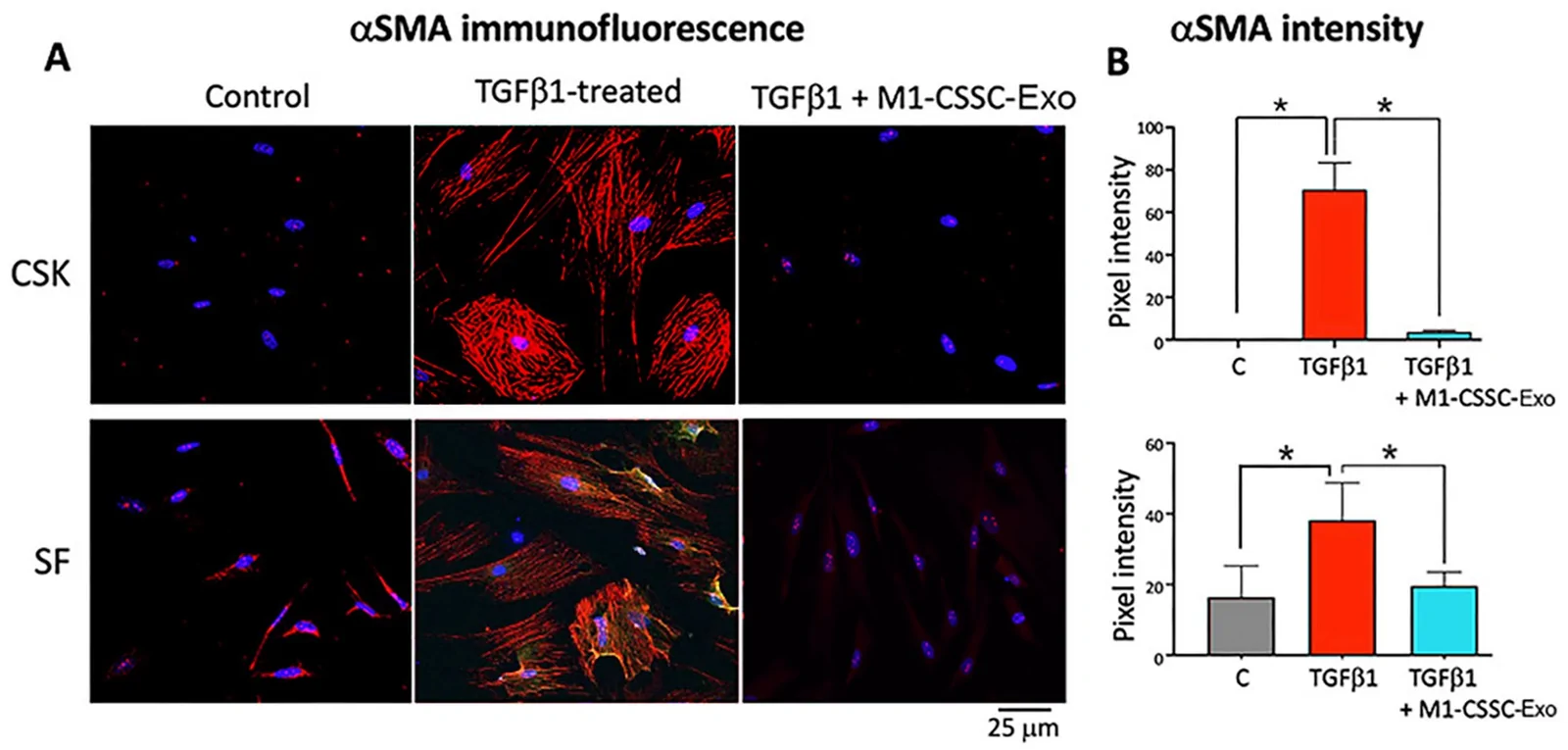
Treatment with M1-stimulated hCSSC-Exo effectively suppressed in vitro fibrosis. (A) hCSKs and hSFs treated with TGFβ1 (10 ng/mL) for 3 days exhibited myoF transition, as indicated by increased αSMA immunofluorescence. Co-treatment with M1-stimulated hCSSC-Exo enriched in *TGFB3* mRNA markedly reduced αSMA expression. (B) Signal densitometry of more than 100 cells demonstrated significant suppression of αSMA expression in both hCSKs and hSFs incubated with M1-stimulated hCSSC-Exo alongside TGFβ1. Data are represented as mean ± SD from triplicate experiment (*P < .05; paired Student’s *t*-test).

Primary hCSKs and hSFs treated with TGFβ1 (10 ng/mL) for 3 days exhibited strong αSMA expression, indicative of myoF differentiation. In contrast, overnight pre-incubation with M1-CSSC-Exo enriched in *TGFB3* mRNA markedly suppressed TGFβ1-induced αSMA expression (Figure 4A). Quantitative fluorescence analysis of cells from three donor corneas (HC579, HC584, and HC670) showed that average αSMA expression was reduced to 5.2% in hCSKs and 52.6% in hSFs relative to TGFβ1-treated controls (*P *< .05; paired Student’s *t*-test) (Figure 4B). These findings demonstrate that M1-CSSC-Exo enriched in *TGFB3* RNA effectively suppress myoF development in both CSKs and SFs.

We then investigated whether this anti-fibrotic effect was mediated primarily by the delivery of *TGFB3* mRNA or by prepackaged TGFβ3 protein within Exo. Utilizing an established Exo Proteinase K (PK) digestion assay (Supplementary Information), surface-associated and encapsulated proteins were degraded, whereas membrane-enclosed RNA was preserved. Primary hCSKs were incubated overnight with hCSSC-Exo, with or without PK pretreatment, and subsequently stimulated with TGFβ1 for 3 days. Immunofluorescence followed by quantification analysis of αSMA-positive cells showed that TGFβ1-induced αSMA expression was significantly reduced in both Exo-treated groups compared with TGFβ1 treatment alone (*P* < .05, Mann-Whitney *U* test) (Supplementary Figure S11). The extent of αSMA reduction was comparable between Exo fractions with and without PK treatment. These findings suggest that the anti-fibrotic activity of hCSSC-Exo is primarily mediated by *TGFB3* mRNA and is insignificantly affected by prepackaged protein.

In addition, treatment with M1-CSSC-Exo reduced cell index of SFs in xCelligence assay, indicative of decreased proliferation and impeded cell size increment that associated with myoF formation (Supplementary Figs. S12A, B). SF migration was also delayed by Exo treatment in an in vitro scratch-wound closure assay (Supplementary Information). The closure rate was 40 ± 12% following treatment with M1-hCSSC-Exo and 44 ± 10% for unstimulated hCSSC-Exo, comparable to that observed with mitomycin C (2 µg/mL; 33 ± 11%). All treatment groups exhibited significantly lower closure rate than untreated controls with 0.5% Exo-free FBS alone (88 ± 16%) (*P* < .05, Kruskal-Wallis test) (Supplementary Figs. S12C, D).

### M1-primed hCSSC-Exo induced scarless healing in mouse corneas

**Figure 5.**
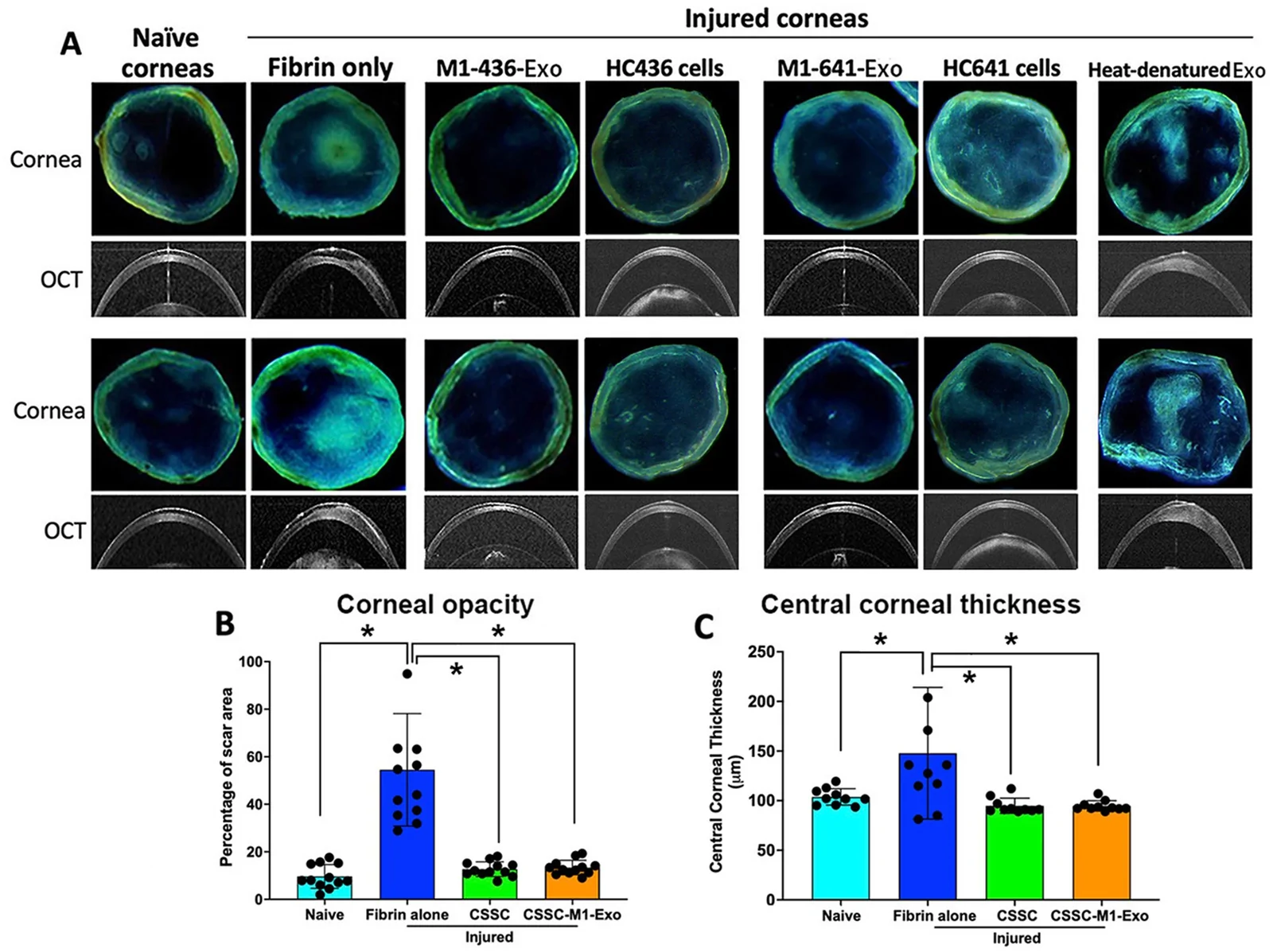
Treatment with M1-stimulated hCSSC-Exo effectively inhibited corneal scarring resembling parental CSSCs. (A) Representative isolated corneas and OCT images at day 10 post-injury and cell treatment with different donor CSSCs (5 × 10^4^ cells) or M1-stimulated CSSC-Exo (10^7^ particles) in a fibrin gel. Heat-denatured Exo lost the scar-inhibitory effect. (B) Both CSSC and Exo treatments reduced the percentages of scarring area (*P < .05, Mann-Whitney *U*-test). (C) A recovery of central corneal thickness to naive levels indicates that both treatments with hCSSC and M1-stimulated hCSSC-Exo alleviated corneal edema associated with scarring (*P < .05, Mann-Whitney *U*-test).

Mouse corneal stromal wounds generated by mechanical ablation were acutely treated with hCSSCs (5 × 10^4^ cells; HC436, HC439 or HC641) or their M1-primed Exo (10^7^ particles) delivered in a fibrin drop (*n* = 6 corneas per group) (Supplementary Figure S3). After 10 days, corneas treated with fibrin alone developed severe scarring, with 53 ± 26% opaque area, whereas naive corneas exhibited <5% opacity (Figure 5A and B). Injured corneas treated with hCSSCs showed markedly reduced opacities (13 ± 3% opaque area), consistent with our previous reports.^7^^,^^9^ Treatment with M1-hCSSC-Exo produced a comparable therapeutic effect, reducing opaque area to 12 ± 4%. Representative images of all treatment groups, including hCSSCs, M1-hCSSC-Exo, fibrin-only controls, and untreated injured controls, are shown in Supplementary Figure S13. Central corneal thickness was also significantly reduced following hCSSC and M1-hCSSC-Exo treatment, reaching levels comparable to naive corneas (*P *< .05, Mann-Whitney *U* test). Moreover, Exo retained their anti-scarring effect after PK digestion, but lost this activity following heat denaturation (Figure 5A; Supplementary Figure S14), indicating that the therapeutic anti-scarring activity was primarily mediated by biologically active *TGFB3* mRNA delivered via Exo, rather than Exo proteins. Collectively, both hCSSCs and M1-primed Exo effectively suppressed corneal scarring following stromal injury.

**Figure 6.**
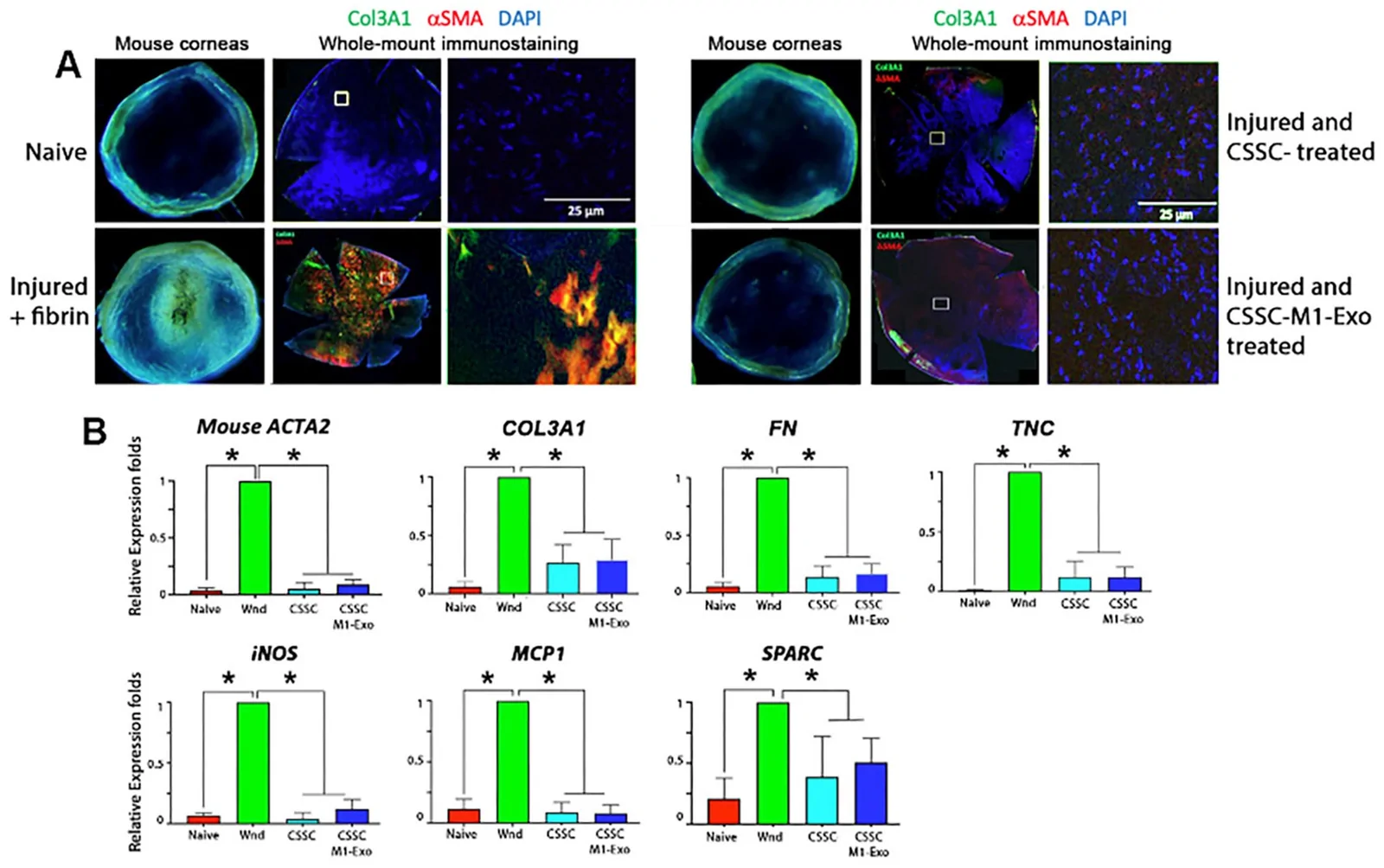
Expression of fibrosis-associated markers in mouse corneas after treatment with M1-stimulated hCSSC-Exo. (A) Representative images showing isolated mouse corneas and immunofluorescence of Col3A1 and αSMA. The treatment with hCSSC (5 × 10^4^ cells) or M1-stimulated hCSSC-Exo (10^7^ particles) in a fibrin gel substantially reduced Col3A1 and αSMA expression which were upregulated in injured corneas. (B) Fibrosis and inflammatory-related gene expression by qPCR. Data are represented as mean ± SD from triplicate experiment. *P < .05, compared to wound control; Mann-Whitney *U* test.

Confocal immunofluorescence revealed strong expression of mouse Col3A1 and αSMA in corneas 10 days after injury and treated with fibrin alone (Figure 6A), confirming fibrosis and scar formation. Treatment with hCSSCs or M1-hCSSC-Exo markedly reduced the expression of both markers. Consistently, qPCR analysis showed significant downregulation of mouse fibrosis-related (*COL3A1*, *ACTA2*, *FN*, *TNC*) and inflammatory genes (*iNOS*, *MCP1*, *SPARC*), compared with the injured group (*P *< .05, Mann-Whitney *U*-test) (Figure 6B). Hence, both hCSSCs and their M1-primed Exo effectively suppress corneal fibrosis, thereby preserving corneal clarity.

### TGFB3 knockdown in hCSSCs attenuated anti-fibrotic effect of M1-hCSSC-Exo

**Figure 7.**
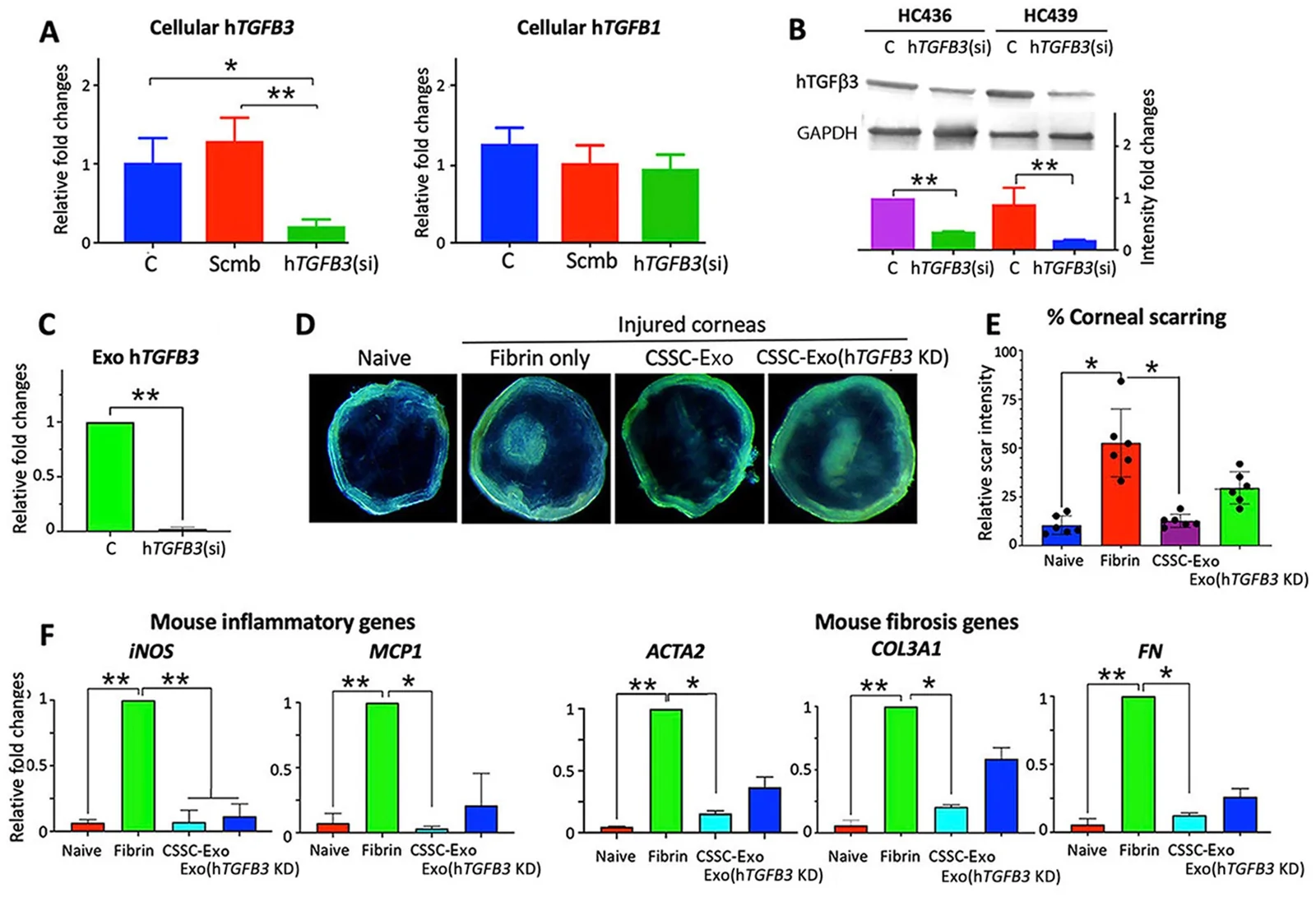
*TGFB3* knockdown (KD) in hCSSCs. (A) Human TGFΒ3(si) transfection specifically reduced *TGFΒ3* expression, with no obvious change in cells transfected with scrambled sequences. *TGFΒ1* expression remained stable with *TGFB3* KD in hCSSCs. (B) Western blot analysis confirmed reduced hTGFβ3 protein expression in TGFβ3(si) transfected cells compared to scrambled controls. Original western blot images were shown in  Supplementary Figure S12. (C) Human *TGFΒ3* RNA levels were significantly reduced in Exo cargo from TGFB3(si)-transfected CSSCs. (D) Mouse anterior stromal injury model. Representative corneal images showing that the scar inhibitory effect of M1-CSSC-Exo treatment was attenuated when TGFB3(si)-Exo were used. (E) Scar area measurement revealed that mouse corneas treated with TGFB3(si)-Exo had greater scarring compared to those with control CSSC-Exo. Both Exo batches were prepared after M1 stimulation. (F) Expression of mouse fibrosis-related genes (*ACTA2*, *COL3A1*, and *FN*) and inflammatory gene (*MCP-1*) remained elevated in corneas treated with TGFB3(si)-Exo, but not with control CSSC-Exo. Data are represented as mean ± SD from triplicate experiment. *P < .05; **P < .01; Mann-Whitney *U*-test.

Knockdown of *TGFB3* in hCSSCs was achieved by siRNA transfection (Supplementary Information). After 48 h, h*TGFB3*(si)-transfected cells exhibited 70 to 80% decrease of *TGFB3* mRNA expression compared with either cells being non-transfected or transfected with scrambled sequences (*P* < .05) (Figure 7A), while *TGFB1* expression was unaffected. Western blot analysis confirmed significant reduction of TGFβ3 protein levels in h*TGFB3*(si)-transfected hCSSCs relative to naive controls (*P *< .05) (Figure 7B; uncropped images in Supplementary Figure S15).

Next, purified Exo fractions were harvested from h*TGFB3*(si)-transfected hCSSCs following stimulation with M1-RAW CM and analyzed by qPCR. *TGFB3* mRNA abundance in h*TGFB3*(si)-Exo was reduced to 1/44^th^ of that detected in Exo from non-transfected cells (*P *< .01; Mann-Whitney *U* test) (Figure 7C).

We further investigated whether hCSSC-Exo exhibited altered scar modulatory effects following *TGFB3* knockdown in the parental cells. Mouse corneas with acute stromal wounds were treated with M1-CSSC-Exo (10^7^ particles; *n* = 6 corneas), which effectively suppressed scar formation at day 10 post-injury (Figure 7D). However, this scar-inhibitory effect was reduced when h*TGFB3*(si)-Exo were administered. The mean scarring area in h*TGFB3*(si)-Exo-treated group was 29.6 ± 7.5% compared with 52.6 ± 15.8% in fibrin-only group, whereas significant reduction of opacity was resulted followed treatment with M1-primed Exo (12.6 ± 5%) (Figure 7E). Consistent with these findings, expression of mouse fibrosis-related genes *ACTA2*, *COL3A1*, and *FN*, and inflammatory marker *MCP-1* remained elevated following treatment with h*TGFB3*(si)-Exo (Figure 7F). These findings indicate that depletion of exosomal *TGFB3* mRNA markedly reduced but did not completely abolish the anti-fibrotic activity of hCSSC-Exo and residual modulatory effects still exists. Other Exo components may contribute to fibrosis modulation and warrants further investigation. Indeed, our pilot analyses of Exo from HC436 cells following h*TGFB3*(si)-transfection revealed no significant changes in the expression of several anti-fibrotic and regenerative microRNAs (hsa-miR-21, 29a, 381) and inflammation-related hsa-miR-155, compared with Exo from naive cells (Figure S16). These findings suggest that *TGFB3* knockdown does not necessarily interfere with Exo cargo composition. In contrast, plasmid-mediated overexpression of *TGFB3* significantly altered multiple exosomal miRNA expression. This observation may reflect the cellular responses to modulated protein synthesis associated with transgene overexpression, which can perturb endosomal trafficking and exosome biogenesis pathways.

### CSSC-Exo enriched with hTGFB3 mRNA exhibited strong anti-scarring effect

**Figure 8.**
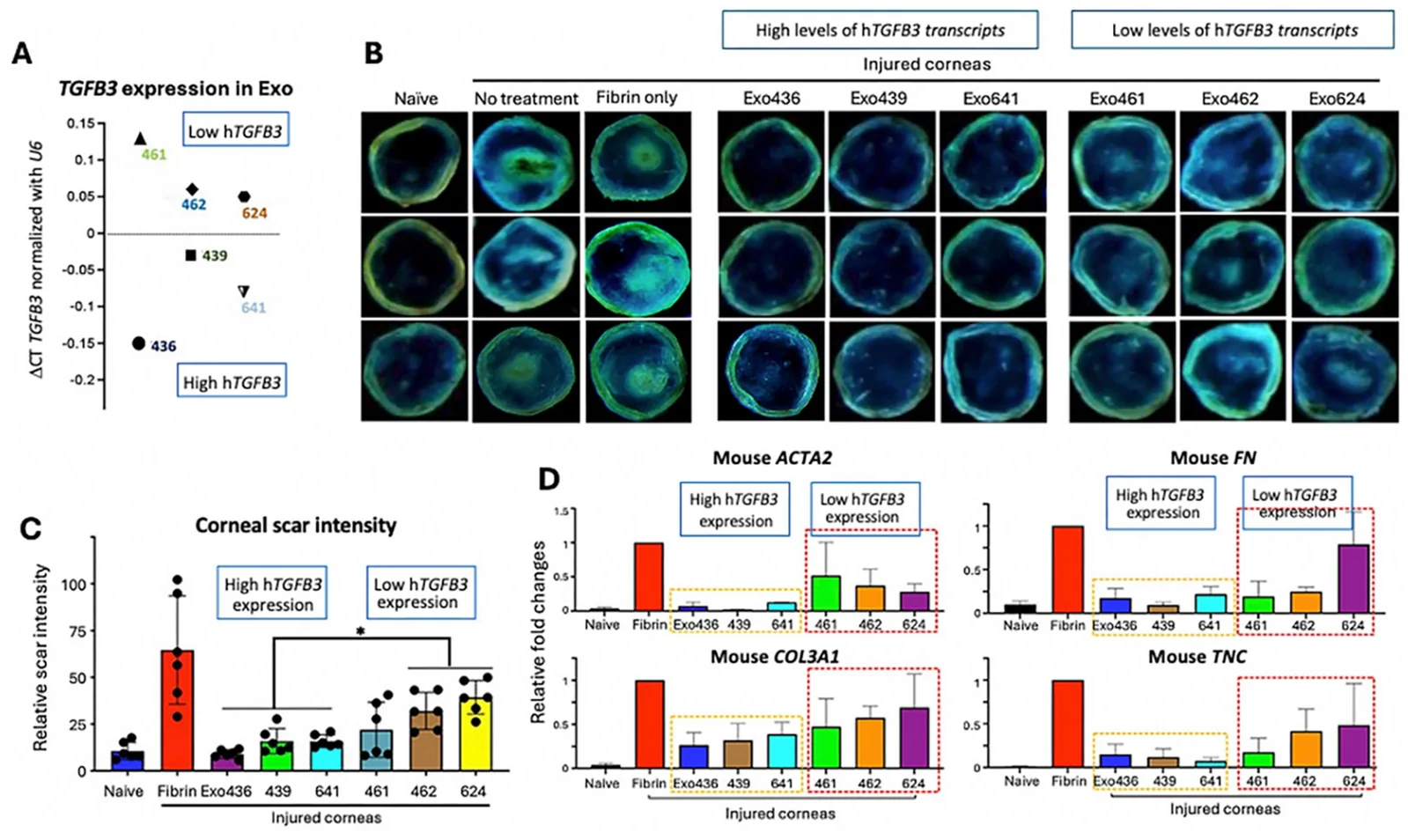
CSSC-Exo enriched in *TGFB3* mRNA transcripts exhibited stronger anti-scarring potency in acute corneal wound treatment. (A) Exo purified from donor CSSCs: Exo436, Exo439, and Exo641 were enriched in *TGFB3* RNA, whereas Exo461, Exo462, and Exo624 carried lower abundance. *TGFB3* RNA levels are represented as ΔCT values normalized to *U6*. (B) At day 10 post-injury and treatment, mouse corneas treated with *TGFB3*-enriched Exo (Exo436, Exo439, and Exo641) demonstrated marked inhibition of scar formation, whereas Exo with lower *TGFB3* content (Exo461, Exo462, and Exo624) were less effective and mild opacities were observed. (C) Quantification of scar intensity confirmed reduced opacities in injured corneas treated with *TGFB3*-enriched Exo (*n *= 6 corneas per treatment group) (*P < .05, Welch’s *t*-test). (D) QPCR analysis of fibrosis-associated gene markers (*ACTA2*, *COL3A1*, fibronectin [*FN*], tenascin-C [*TNC*]). All genes were upregulated in fibrin-only treated injured corneas compared with naive controls. Treatment with Exo reduced the upregulated levels, with *TGFB3*-enriched Exo showing stronger anti-fibrotic effect than Exo with low abundance (*n *= 6 corneas per group).

Exo fractions from six hCSSC batches (HC436, HC439, HC461, HC462, HC624, and HC641) were analyzed for full-length *TGFB3* mRNA expression. ΔCT values normalized to U6 showed that Exo fractions from HC436, HC439, and HC641 contained higher *TGFB3* mRNA abundance than those from HC461, HC462 and HC624 (Figure 8A).

Topical hCSSC-Exo treatment of mouse acute corneal wounds (*n* = 6 corneas per group) generally suppressed opacity formation compared with untreated injured corneas or those treated with fibrin alone. Particularly, corneas treated with *TGFB3* mRNA-enriched Exo (ie, Exo436, Exo439, and Exo641) exhibited greater opacity inhibition than those receiving Exo with lower RNA abundance (ie, Exo461, Exo462, and Exo624) (Figure 8B). The mean scar intensity was 13.6 ± 5.3 pixels in corneas treated with *TGFB3* mRNA-enriched Exo, compared with 31.1 ± 12.7 pixels in corneas receiving Exo with lower RNA abundance (*P *< .05, Welch’s *t*-test) (Figure 8C). Consistent with this observation, qPCR analysis showed significantly upregulated *FN*, *COL3A1*, *TNC*, and *ACTA2* in fibrin-treated injured corneas relative to naive controls (Figure 8D). Treatments with Exo containing either high or low h*TGFB3* RNA levels suppressed injury-induced expression of these genes; particularly, Exo *TGFB3* mRNA-enriched Exo produced stronger suppressive effects. Together, these results demonstrate a positive association between *TGFB3* mRNA abundance in Exo cargo and anti-scarring efficacy, supporting a functional role for hCSSC-Exo in reducing corneal fibrosis.

### Elevated hTGFβ3/β1 ratios inhibited myofibroblast differentiation from hSFs and hCSKs

**Figure 9.**
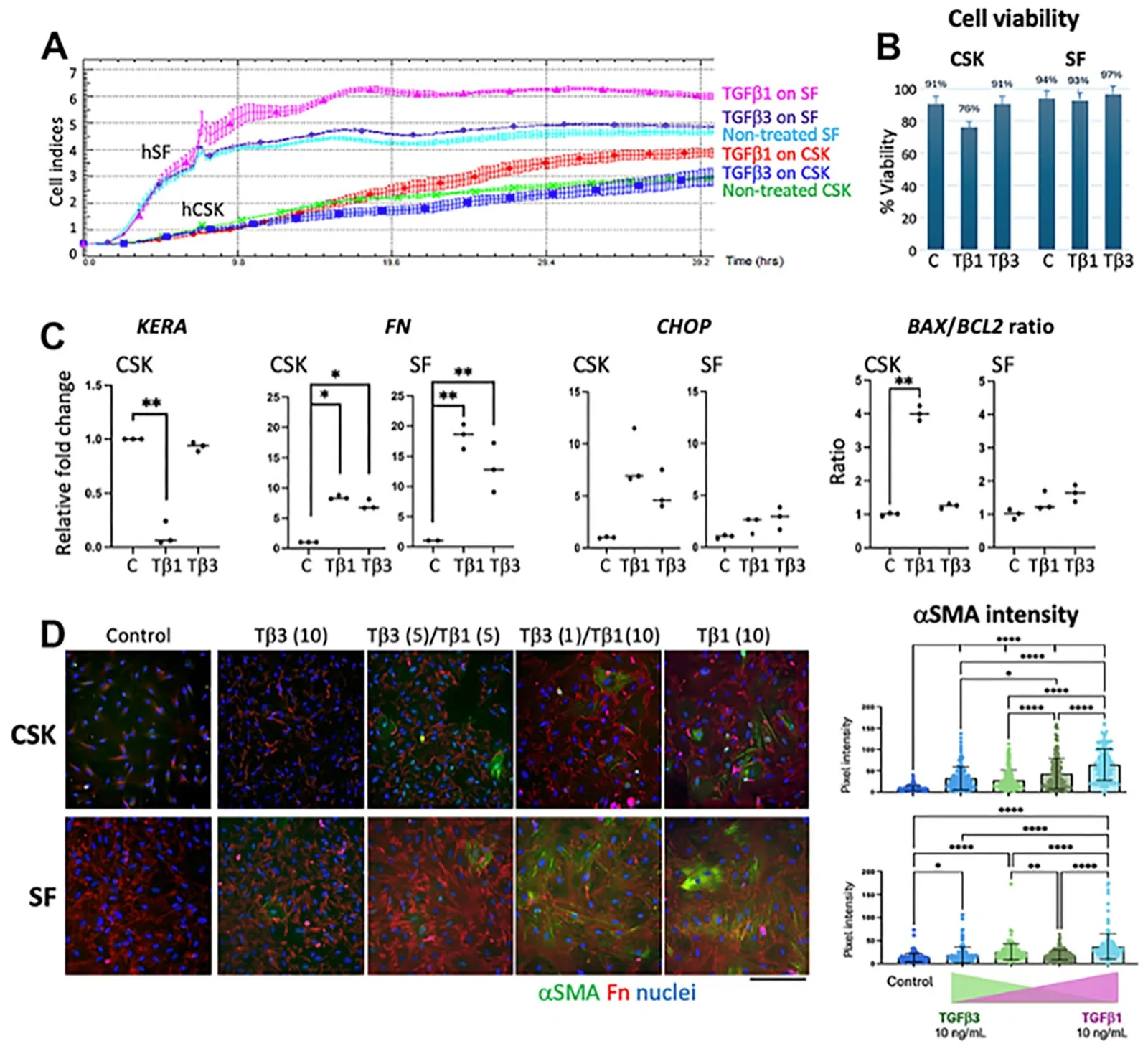
Effects of varying TGFβ3/β1 ratios on corneal stromal cells. (A) xCelligence assay to study cell growth of hCSKs and hSFs treated with TGFβ1 (10 ng/mL) or TGFβ3 (10 ng/mL), compared with controls. TGFβ1 enhanced cell growth or size changes whereas TGFβ3 treatment had minimal effects on hCSK or hSF growth. (B) Trypan blue dye exclusion assay to assess cell viability changes after TGFβ1 or TGFβ3 treatment for 48 h. TGFβ1-treated hCSKs had reduced viability while SF viability remained. No change was observed for TGFβ3 treatment. (C) QPCR analysis to evaluate the effects of TGFβ1 or β3 on the expression of keratocyte-specific gene keratocan (*KERA*), fibroblast marker fibronectin (*FN*), and apoptosis indicators C/EBP homologous protein (*CHOP*) and *BAX*/*BCL2* ratio. (D) Representative confocal images showing immunofluorescence for αSMA and FN in cells treated with varying TGFβ3/β1 ratios for 48 h. The numbers in brackets represent growth factor dosage at ng/mL. Full-sized images are shown in Supplementary Figure S14. Signal intensity was quantified using ImageJ analysis with at least 200 cells per group. Data are represented as mean ± SD from triplicate experiment. *P < .05, **P < .01, ***P < .001, ****P < .0001; paired Student’s *t*-test.

Paired cultures of hCSKs and hSFs (*n* = 3 donor corneas) were treated with varying ratios of recombinant hTGFβ1 and β3 (total cytokine concentration up to 10 ng/mL) for 4 days. Because TGFβ proteins have a short half-life,^32^ fresh media containing cytokines were replenished daily to maintain the biological activity. From triplicated experiments, xCelligence showed that TGFβ1 increased cell indices in both hCSKs and hSFs, consistent with enhanced cell growth or spreading associated with myoF generation (Figure 9A). In contrast, TGFβ3 did not alter cell indices in either cell type, similar as untreated controls. Trypan blue exclusion assay further showed reduced viability in TGFβ1-treated hCSKs (76 ± 3.8%) compared with untreated control (91 ± 4.5%), whereas hSF viability remained unchanged (93 ± 4.7% versus 94 ± 4.7% in controls) (Figure 9B).

To determine whether TGFβ isoforms differentially regulate stromal cell phenotypes, we assessed expression of CSK and SF markers by qPCR. In hCSKs, *KERA* expression was significantly downregulated by TGFβ1 (89 ± 9% reduction relative to untreated controls, *P* < .01, Mann-Whitney *U* test), whereas TGFβ3 produced insignificant reduction (6.8 ± 3.9%) (Figure 9C). As expected, *KERA* expression was undetectable in SFs irrespective of treatment. *FN* expression was significantly induced by both TGFβ1 and TGFβ3 in hCSKs (8.4-fold and 7.2-fold, respectively; *P *< .05). In hSFs, FN upregulation was greater following TGFβ1 treatment (18.4 ± 2.1-fold) than TGFβ3 (13 ± 4-fold) compared with naive controls (both *P *< .01) (Figure 9C). These results suggest that hCSKs had more pronounced phenotypic modulation in response to TGFβ1 than TGFβ3, while SFs had comparatively modest changes.

Increased cell death following TGFβ1 treatment in hCSKs might account for their reduced viability. Expression of C/EBP homologous protein (*CHOP*), a regulator of cell cycle arrest and apoptosis,^33^ was moderately increased in hCSKs following treatment with either TGFβ1 or β3 but showed only minimal induction in hSFs (Figure 9C). Notably, TGFβ1-treated hCSKs exhibited a significantly higher *BAX/BCL2* ratio (8.4 ± 2.7-fold above untreated controls) than TGFβ3-treated cells (5.4 ± 1.9-fold); whereas no obvious changes were detected in hSFs. These findings indicate that TGFβ1 preferentially triggered apoptosis in hCSKs, a response not observed with TGFβ3, while hSF viability was unaffected.

Finally, we examined how TGFβ3/β1 ratios influence myoF differentiation by immunofluorescence for αSMA. Quantitative signal analysis showed that hCSKs treated with TGFβ1 only displayed a moderate increase of αSMA expression (Figure 9D). In contrast, raising the proportion of TGFβ3 relative to TGFβ1 (increasing TGFβ3/β1 ratio) progressively reduced αSMA expression. Cells treated solely with TGFβ3 did not express αSMA, similar as untreated controls. Analysis of more than 200 cells per group revealed a decreasing mean pixel intensity of αSMA as TGFβ3/β1 ratios increased, compared with cells treated with TGFβ1 only (Figure 9E). Similarly, hSFs exposed to lower TGFβ1 concentrations (higher TGFβ3/β1 ratios) also exhibited decreased αSMA expression (Figure 9D; Supplementary Figure S17). Collectively, these findings demonstrate that elevated TGFβ3/β1 ratios maintain the native CSK phenotype while suppressing myoF differentiation in both hCSKs and hSFs. In contrast, TGFβ1 adversely affected CSK viability by promoting apoptosis and loss of keratocyte phenotype.

## Discussion

In this study, we demonstrate that hCSSC-Exo transport protein-coding *TGFB3* mRNA and promote scar-free corneal healing. Transiently exposure of hCSSCs to non-lethal stressors, including pro-inflammatory M1 and nutrient deprivation, increased *TGFB3* mRNA expression in both cells and their Exo. In vitro, hCSKs and hSFs that internalized M1-primed hCSSC-Exo produced TGFβ3 proteins, which suppressed TGFβ1-induced myoF differentiation. Topical administration of hCSSC-Exo also reduced corneal opacity following acute injury in mice. hCSSC-Exo enriched with *TGFB3* mRNA induced better scar-free healing relative to Exo with lower RNA abundance. Collectively, these findings demonstrate the therapeutic potential of M1-primed hCSSC-Exo as a cell-free regenerative therapy for preventing corneal fibrosis.

Our previous study identified TGFβ3 as a key regenerative cytokine produced by hCSSCs.^11^ Following hCSSC treatment of mouse corneal stromal wounds, hTGFβ3 expression was upregulated during early time points (1 and 3 days), along with reduced inflammatory and fibrotic marker expression. Specific knockdown of TGFβ3 in hCSSCs diminished this anti-scarring efficacy, resulting in intense scarring. These findings indicate that hCSSCs promote corneal regeneration through paracrine TGFβ3 signaling. However, active TGFβ proteins have a short half-life of only minutes,^34^ although latent protein complexes remain stable for only a few hours.^35^ Such transient availability is unlikely to account for the sustained therapeutic benefits of CSSC treatment, suggesting that additional mechanisms, such as prolonged cytokine production mediated by specialized depots may contribute to scar suppression.

TGFβ signaling is critical for tissue healing.^36^ In corneas, following injury, TGFβ1 and β2 are rapidly released from the damaged epithelium and basement membrane, activating quiescent CSKs to become proliferative SFs and αSMA-positive myoFs.^3^ These cells deposit excessive and disorganized ECM, leading to opacity that compromises corneal transparency.^37^^,^^38^ These pro-fibrotic pathways could be redirected toward a scar-reducing or scarless phenotype when higher levels of anti-fibrotic cytokines relative to pro-fibrotic molecules are established.^39^ Various studies have shown that TGFβ3 exhibits anti-fibrotic and regenerative properties.^40^^,^^41^ Whereas TGFβ1 promotes fibrotic gene expression and pathological ECM deposition, TGFβ3 supports non-fibrotic stromal matrix remodeling, preserves collagen fibril organization, and maintains stromal architecture.^14^^,^^15^ In studies with fetal tissues and adult oral mucosa, the balance between TGFβ3 and TGFβ1 is a critical determinant of healing outcome.^12^^,^^13^^,^^42^^,^^43^ A high TGFβ3/β1 expression ratio is consistently associated with scar-free repair, whereas a low ratio is linked to fibrotic healing. Therefore, strategies that increase TGFβ3/β1 ratio soon after injury may suppress fibrosis and redirect tissue healing toward regenerative scar-free repair.

In this study, we found that hCSSCs upregulated *TGFB3* expression in response to transient inflammatory stress, consistent with observations in tissue-specific MSCs.^18^ This adaptive response enables hCSSCs to withstand the hostile wound-related environment while enhancing their regenerative capacity. We further demonstrate that hCSSCs exert paracrine anti-fibrotic effect through Exo carrying *TGFB3* mRNA, which was upregulated following M1 pro-inflammatory stimulation. The integrity of exosomal *TGFB3* mRNA was verified by PCR amplification of the 5’UTR, 3’UTR, and full-length coding sequence, respectively, with cDNA synthesized using oligo-dT primers that bind to the polyadenylated end of mRNA molecules (Supplementary Figure S2). If mRNA is intact full-length, reverse transcription from poly(A) tail goes on uninterruptedly, generating cDNA, which yields similar band intensity of 5’ and 3’UTR amplicons.^44^ For fragmented RNA, PCR with truncated oligo(dT)-primed cDNA fragments produces less 5’UTR end products. Our results showed a consistent 5′:3′-UTR amplicon ratio of approximately 1, indicating that Exo carry intact full-length *TGFB3* mRNA capable of being transferred to recipient cells.

After internalizing these Exo, recipient cells become depots in translating *TGFB3* mRNAs into functional TGFβ3 protein. As shown in our cultured stromal cell model, the biosynthesis of hTGFβ3 protein was visualized by immunofluorescence. In vitro fibrosis assay further demonstrated that uptake of Exo by hCSKs and hSFs markedly suppressed TGFβ1-induced fibrotic gene upregulation. Importantly, this anti-fibrotic activity was preserved following proteinase K treatment of Exo, suggesting that the observed effects are predominantly mediated by Exo-delivered *TGFB3* mRNA and are largely independent of prepackaged TGFβ3 protein or trace amounts of TGFβ3 protein carried over via Exo.

We hypothesize that Exo-mediated *TGFB3* mRNA transfer amplifies anti-fibrotic signaling, thereby promoting tissue repair. In vivo, M1-CSSC-Exo treatment suppressed inflammatory and fibrotic responses in injured mouse corneas. This paracrine mechanism may produce sustained healing effects by shifting the wound environment toward resolution of inflammation and fibrosis while restoring tissue homeostasis. Our recent studies also demonstrated that hCSSC-Exo carry anti-fibrotic microRNAs, suggesting that multiple Exo cargo act communally to promote scarless wound healing.^23^^,^^25^

To investigate the impact of TGFβ3/β1 ratio, hCSK (quiescent) and hSF (proliferative) cultures were treated with varying ratios of recombinant hTGFβ1 and β3. We observed that low TGFβ3/β1 ratios led to increased αSMA expression while higher ratios reversed this effect. Additionally, FN expression was upregulated in both hCSKs and hSFs following treatment with either TGFβ1 or TGFβ3. Notably, only hCSKs treated with TGFβ1 showed evidence of apoptosis, as indicated by upregulated *CHOP* expression and *BAX*/*BCL2* ratio. These findings support our hypothesis that higher TGFβ3/β1 ratios are beneficial for maintaining stromal cell viability and promoting a less fibrotic phenotype.

The anti-fibrotic property of hCSSC-Exo was examined in vivo through modulating visible scarring in acute corneal wounds. The treatment significantly reduced tissue expression of fibrotic and inflammatory genes. Our findings also establish a link between the regenerative properties of CSSC-Exo and their cargo content. Notably, hCSSC-Exo enriched with *TGFB3* mRNA exhibited better scar-free healing compared to Exo with lower abundance and even knockdown of *TGFB3*. Importantly, hCSSC-Exo were delivered in a fibrin vehicle, which gradually degrades over time and generates products known to promote fibrotic responses.^45^ Consistent with this, fibrin-only sham-treated corneas developed pronounced fibrosis and scarring. Despite this potentially pro-fibrotic environment, hCSSC-Exo delivered in the same fibrin vehicle markedly reduced corneal scarring, demonstrating that their anti-fibrotic activity outweighs the effects of fibrin degradation products while reducing injury-induced inflammation and fibrosis.

Summarized from various studies, hCSSC-Exo carrying regenerative molecules represent a promising next-generation, cell-free approach for fibrosis therapy. This strategy is supported by several key findings: (1) hCSSCs producing Exo enriched with miR29a exhibit significant scar-inhibitory effects in corneal wound healing^23^; (2) MSC-Exo can be engineered to deliver miR-29 family cargo, inhibiting TGFβ/Smad signaling, downregulating pro-fibrotic genes and reducing inflammation and aberrant ECM remodeling in both cutaneous and corneal injury models^46^^,^^47^; (3) scar-relieving and regenerative healing, as opposed to fibrotic repair, in ocular and cutaneous tissues can be achieved by modifying the TGFβ3/β1 balance,^11^^,^^15^^,^^22^ making TGFβ3-loaded Exo appealing vehicles for modulating canonical TGFβ pathways; and (4) in this study, M1-hCSSC-Exo transport protein-coding *TGFB3* mRNA, which, following stromal cell uptake, generate numerous depots to produce functional TGFβ3 protein, leading to amplifying the anti-fibrotic and regenerative signals. Additional reports have shown that M1-primed MSCs reduce fibrotic activation by reprogramming the metabolism of inflammatory macrophages and fibroblasts across glycolytic, lipid, and amino acid pathways, including the PI3K/AKT/mTOR and STAT signaling axes.^48–51^ Collectively, these findings support our hypothesis that hCSSC-Exo can suppress corneal inflammation and fibrosis through altering stromal cell metabolism and attenuating abnormal ECM synthesis and deposition.

A limitation of this study is that treatment efficacy of CSSC-Exo was evaluated in an acute corneal wound model, which does not fully recapitulate the clinical setting of chronic and preexisting scarring. Therefore, the treatment outcomes observed in this study may not be directly translatable to patient situations, warranting further investigations with clinically relevant models. A key advantage of evaluating CSSC-Exo’s therapeutic effect using an acute injury model is that fibrosis and opacity occurs from healthy tissue and follows a predictable temporal course, enabling a controlled assessment of treatment effects. In contrast, chronic wounds are biologically heterogeneous with variable pathological features, inflammatory states, and healing responses, all of which can influence treatment outcomes.^52^ In our previous studies, assessment within 10 to 14 days following injury was sufficient to determine treatment efficacy based on scar outcomes.^7^^,^^9^^,^^10^ These data provide a robust proof-of-concept demonstration of the therapeutic anti-fibrotic efficacy. Another limitation is the lack of direct evidence showing newly synthesized hTGFβ3 protein in mouse corneas after hCSSC-Exo treatment. This is primarily due to the absence of antibodies capable of reliably distinguishing between human and mouse TGFβ3 protein. Similar to other members of TGFβ family, TGFβ3 is highly conserved across species, with human and mouse *TGFB3* mRNA exhibiting 100% sequence homology.^53^ Future studies using alternative animal models, such as rabbits, will evaluate this in vivo molecular translation.

Additionally, pro-inflammatory priming of hCSSCs was achieved using M1-RAW-CM, which is not compatible with Good Manufacturing Practice (GMP) requirements, and, hence, limits clinical translation. Future studies should evaluate GMP-compliant alternatives, such as recombinant interferon-γ (IFNγ) or tumor necrosis factor-α (TNFα), to enhance hCSSC therapeutic potency while facilitating scalable manufacturing.^19^^,^^54^ Moreover, hCSSCs were exposed to M1-RAW-CM for only 48 hours as a transient stress stimulation and Exo were collected. We selected this transient priming strategy to mimic the early inflammatory phase of wound healing, during which macrophages initially adopt a pro-inflammatory phenotype before transiting to a regenerative M2 state. Although prolonged inflammatory stimulation may alter hCSSC phenotypes and warrants future investigation, it is less representative of the transient inflammatory environment that characterizes the acute wound response.

Also, we acknowledge that the concentrations of recombinant TGFβ1 and β3 used in our in vitro fibrosis assay are at higher ends of the physiological range. Because endogenous concentrations of TGFβ isoforms in corneal tissues have not been well characterized, and many studies used 10 ng/mL TGFβ to induce fibrotic and stress-related responses in corneal cells,^55–58^ we adopted a similar dosage framework for our exploratory dose-response experiments. This concentration reliably induced myoF formation, allowing us to investigate the biological effects of varying TGFβ3/β1 ratios. Future studies should evaluate the effects of TGFβ within physiological relevant levels to avoid potential risk of receptor saturation and activation of non-physiological signaling crosstalk.^59^

Furthermore, our in vivo study evaluated a single Exo dose (10^7^ particles), consistent with our reports.^7^^,^^25^^,^^60^ Future dose-response studies are warranted to determine the minimal effective dose and optimal therapeutic window in achieving maximal efficacy. Although assessing multiple dosing may enhance the therapeutic efficacy and provide valuable information about dose-response relationships; our current delivery strategy requires epithelial debridement to expose the stroma for Exo treatment. Repeated debridement could impair epithelial and basement membrane regeneration, and may lead to unforeseen adverse effects. Alternative delivery formats, like stromal injection and liquid eye-drop formulation, hydrogel encapsulation, surface engineering to enhance Exo retention and biodistribution, as well as long-term safety should be evaluated before clinical translation. Finally, comprehensive multi-omics analyses, including transcriptomic, proteomic, metabolomic, and small RNA profiling, will be valuable for identifying additional components in hCSSC-Exo that contribute to scarless healing and for elucidating mechanisms underlying the regenerative effects.

## Conclusions

Cell-free therapy using M1-primed hCSSC-Exo represents a promising alternative to corneal transplantation for treating corneal scarring and opacity. By delivering bioactive cargo, including *TGFB3* mRNA and anti-fibrotic microRNAs, these Exo suppress tissue inflammation and fibrosis while promoting stromal repair.

## Supplementary Material

szag079_Supplementary_Data

## Data Availability

All data are included in the text and supplementary materials. Data details are available from the corresponding author on request.
