# Supplementary material for: M1-primed human corneal stromal stem cell-derived exosomes delivering protein-coding transforming growth factor-β3 RNA promote corneal regeneration
Source: Stem Cells Transl Med. 2026 Sep 4;15(9):szag079. doi: 10.1093/stcltm/szag079 (PMC13543683; doi:10.1093/stcltm/szag079)
Supplement: Supplementary file 1 [file szag079_supplementary_data.docx]

***Supplementary Information***

**M1-Primed Human Corneal Stromal Stem Cell-Derived Exosomes Delivering Protein-Coding Transforming Growth Factor-β3 RNA Promote Corneal Regeneration**

*Running title:* Human CSSC-exosomes deliver TGFβ3 RNA

Mithun Santra^1#^, Elizabeth Rubin^1⊕^, Syeda R. Ali^1^, Moira L. Geary^1^, Christine Chandran^1∂^, Julia T. Coelho^1^, Ella J. Dewald^1^, Xi Cheng^2^, Martha L. Funderburgh^1^, Deepinder K. Dhaliwal^1^, Xin D. Gao^2,3,4^, Vishal Jhanji^1^, Gary H. F. Yam^1,5^ *

^1^Corneal Regeneration Lab, Department of Ophthalmology, University of Pittsburgh School of Medicine, Pittsburgh, PA 15219, USA

^2^Department of Ophthalmology, University of Pittsburgh School of Medicine, Pittsburgh, PA 15219, USA

^3^Department of Bioengineering, University of Pittsburgh, Pittsburgh, PA 15219, USA

^4^Institute for Synthetic Biology, University of Pittsburgh, Pittsburgh, PA 15219, USA

^5^McGowan Institute for Regenerative Medicine, University of Pittsburgh, Pittsburgh, PA 15219, USA

\# Present affiliation: Department of Ophthalmology, Postgraduate Institute of Medical Education and Research, Chandigarh 160012, India

^⊕^ Present affiliation: Perelman School of Medicine, University of Pennsylvania, Philadelphia, PA 19104, USA

^∂^ Present affiliation: Johns Hopkins Bloomberg School of Public Health, Johns Hopkins University, Baltimore, MD 21205, USA

***** Correspondence:

Gary H. F. Yam, Ph.D.

Department of Ophthalmology, University of Pittsburgh School of Medicine

Mercy Vision Institute, 1622 Locust St., Pittsburgh, PA 15219, USA.

E-mail: [yamg@pitt.edu](mailto:yamg@pitt.edu)

**Materials and Methods**

***1. Donor corneas***

Research-grade corneas from de-identified donors were procured from the Center for Organ Recovery and Education (protocol #161; approved on Sep 12, 2024), Pittsburgh, PA and Eversight Eye Bank. The study was approved by Human Stem Cell Research Oversight (Protocol# 202300049; approved on Feb 17, 2025) at the University of Pittsburgh. Donors had no history of ocular diseases or cancer and were negative for transmissible diseases including human immunodeficiency virus, hepatitis virus B and C, and syphilis. The corneas were free from prior wounds and had endothelial cell count greater than 2,500 cells per mm^2^. They were harvested by trained eye bank technicians under sterile conditions. The time from donor death to preservation in Optisol-GS (Bausch & Lomb, Rochester, NY) was less than 12 hours and corneas were utilized within 14 days. A total of 21 donor corneas (mean donor age 48.5±15.5 years old and a male/female ratio 10:11) were used (Supplementary Table S1).

***2. Primary stromal cell cultures***

***2.1 Human corneal stromal stem cells (hCSSCs)***

After removal of corneal epithelium and endothelium, the anterior limbal stroma (0.5 to 1 mm width and 0.2 μm depth) was isolated and digested with 0.1% (weight/volume) collagenase A (Roche, Indianapolis, IN) for 6 to 8 hours at 37°C, per our published protocol.^1-3^ After filtering through a cell strainer (40 μm pore size, Corning, NY), single cell suspension was collected, washed, and cultured on polystyrene tissue culture surface (TCPS) pre-coated with FNC mix (serum-free reagent with fibronectin, collagen, and albumin; Athena Enzyme Syst., Baltimore, MD) using stem cell growth medium (JM-H) supplemented with 2% (vol/vol) pooled human serum (Innovative Res, Novi, MI). Clonally expanded cells were propagated to passage 3 (P3) for the assessment of stem cell marker expression.

***2.2 Human corneal stromal keratocytes (hCSKs) and stromal fibroblasts (hSFs)***

The central stroma was digested with 0.1% (weight/volume) collagenase A for 6-8 hours at 37°C. The lysate was passed through cell strainer and single cell suspension was collected, washed, and cultured on collagen I-coated TCPS using ERI protocol added with 0.5% heat-inactivated fetal bovine serum (FBS, Gibco, Thermo Fisher Sci, Waltham, MA) (0.5SERI medium) to propagate hCSKs until P4.^4,5^ Cells were placed in a serum-free ERI condition for a week to generate quiescent hCSKs, which were assessed for marker expression specific to keratocytes including keratocan (KERA) and aldehyde dehydrogenase 3A1 (ALDH3A1). SFs were generated by culturing CSKs with 5% FBS for two passages. SF phenotype was confirmed by COL3A1 and FN expression.

***3. Exosome preparation and quantification***

The protocol strictly adhered to the guidelines of 2021 MISEV (Minimal Information for Studies of Extracellular Vesicles).^6^ hCSSCs were propagated to about 60% confluence, washed, and maintained in serum-free condition supplemented with insulin-transferrin-selenium mix (Thermo Fisher). Conditioned media (CM) was collected on day 2 and 4. Samples were spun at 400g for 5 min to remove cellular debris. The supernatant was further centrifuged at 12,000g for 15 min at 4°C to eliminate apoptotic bodies.^7,8^ Supernatant was concentrated to about 1/20^th^ of the original volume using MicroCon centrifugal filter (YM-10, Millipore Sigma, Burlington, MA). Exosomes were precipitated by mixing 1:1 vol/vol with Total Exosome Purification Reagent (Invitrogen) for overnight at 4°C. After centrifugation at 12,000g for 1h at 4°C, the pellet was resuspended in sterile PBS added with 50 mM trehalose (Sigma-Aldrich) (PBS+Th) with gentle agitation at 4°C overnight. The Exo suspension was ultra-centrifugated at 100,000g for 2h at 4°C (Optima L-90K Ultracentrifuge, Beckman Coulter; SW32Ti rotor). The Exo-enriched pellet was then resuspended in PBS+Th overnight. After another centrifugation at 100,000g for 2h, the final Exo fraction was resuspended in sterile PBS+Th.

***4. Exosome labelling and cellular uptake***

CSSC-Exo suspension was incubated with Carboxyfluorescein succinimidyl ester (CFSE, 20 μM; Thermo Fisher) for 2h at 37°C.^8^ After buffer washes followed by centrifugation for two times, CFSE-labeled Exo (25 μg protein) were applied to hCSK and hSF cultures, respectively, for 4h. After extensive PBS washes, cells were fixed with ice-cold absolute ethanol for 20 min. The nuclei were counterstained with 4’,6-diamidino-2-phenylindole (DAPI) for 5 min, and cells were examined using confocal microscopy (Fluoview 1200, Olympus, Center Valley, PA).

***5. Detection of full-length and protein-coding hTGFB3 RNA transcripts***

Exo RNA was reverse transcribed with oligo d(T)_23_ VN primer (New Engl Biolabs, Ipswich, MA) and cDNA was amplified for protein-coding human *TGFB3* mRNA (GenBank Accession No. [NM_001329939.2](https://www.ncbi.nlm.nih.gov/entrez/viewer.fcgi?db=nucleotide&id=1889694561)) using specific primers: forward: 5’-ATGAAGATGCACTTGCAAAGGGC-3’ and reverse: 5’-TCAGCTACATTTACAAGACTTCACCAC-3’ to generate amplicon from the start codon (ATG) to the stop codon (TGA) (Supplementary Fig. S2). Alternatively, 5’UTR was detected using specific primers: forward: 5’-AAAAGGCCTAGAGGTTCGGG and reverse 5’**-**TCTCGTCTTCATTGGCTGGG; whereas 3’UTR with primers: 5’- TGGACACTTGGTTAGACGCC and reverse 5’-GAACACAGGGTCTTGGAGGG. After amplification using a hot-start PCR kit (New Engl Biolabs), the products of protein-coding human *TGFB3* mRNA (1,239 bp), 5’UTR (363 bp), and 3’UTR (115 bp) was assessed by 0.8-1.5% agarose gel electrophoresis.

***6. Preparation of M1 pro-inflammatory and M2 anti-inflammatory RAW condition media (CM)***

Mouse macrophage RAW264.7 cells (American Type Cell Collection, Manassas, VA, USA) at passage 9 were seeded at a density of 10^4^ cells/cm^2^ in DMEM/F12 medium supplemented with 10% heat-inactivated fetal bovine serum (FBS, Gibco). When cells reached 60% confluence, the culture was treated with lipopolysaccharide (LPS, 25 ng/mL, Sigma-Aldrich) for 24h to undergo M1 polarization. Alternatively, RAW culture was treated with interleukin-4 (IL4, 20 ng/mL, Sigma-Aldrich) for 24h to induce an M2 phenotype. After PBS washes, serum-free DMEM/F12 medium was added to these cultures. After 24h, conditioned medium (CM) was collected from M1, M2, as well as untreated RAW (M0) cultures, and concentrated by centrifugation using MicroCon filters (YM-10 membrane, Millipore) at 12,000 g at 4°C until the volumes were reduced to approximately 1/20^th^ of the original volumes, resulting in CM concentrate (CMconc). Total protein content of CMconc was quantified using the Pierce BCA Protein Assay kit (Thermo Fisher Scientific). For M1 (LPS-treated), M2 (IL4-treated), and M0 (non-treated) RAW cells, total RNA was isolated for the expression analysis of inflammatory markers, including mouse MCP1 and iNOS to confirm M1 transition; mouse ARG1 and PTGS1 to assess M2 phenotype (Supplementary Figure S4).

***7. Cell stress induction***

***7.1 Pro-inflammatory M1 stimulation***

Mouse macrophage RAW264.7 cells were cultured in DMEM/F12 with 10% FBS. At 60% confluence, cells were treated with liposaccharides (LPS, 20 ng/mL, Sigma-Aldrich, St Louis, MO) for 24h to induce M1 polarization, following our reported protocol.^9^ After washes, M1 RAW cells were cultured in DM+ for 24h. CM was collected and concentrated to 1/20^th^ original volume as described above (M1-RAW-CM) (Supplementary Fig. S4). RAW cells without LPS treatment were processed similarly to obtain M0-RAW-CM. The cultures of hCSSC, hCSK, and hSF at 60% confluence were added with RAW-CM (M1 and M0, respectively; 100 μg protein/mL) for 48h. Controls were cells cultured in DM+. Cells were collected for total RNA isolation. The expression of TGFβ isoforms were evaluated using qPCR and specific primers (Supplementary Table S2).

***7.2 Nutrient deprivation (ND)***

At about 60% confluence, hCSSCs were replenished with serum-free DMEM/F12 supplemented with 1% ITS mix (DM+) for 48h. Cells were collected and lysed in RLT buffer for total RNA extraction. The expression levels of TGFβ isoforms were evaluated using quantitative PCR (qPCR).

***7.3 H_2_O_2_-mediated oxidative stress induction***

We followed a reported protocol for H_2_O_2_-mediated oxidative stress induction with modifications.^10^ Cells were treated with 20 μM H_2_O_2_ for 6 days with medium having fresh H_2_O_2_ replenished every day. Cells were lysed in RLT buffer, and total RNA extracted for gene expression analysis with unfolded protein response (UPR) and oxidative stress markers. The expression of TGFβ isoforms was assessed for fold change difference normalized with untreated controls.

***7.4 Transient heat shock stress induction***

Following reported protocols,^11,12^ the culture medium was pre-warmed to 40°C before adding to cell culture and incubated at 40°C to induce heat shock for 90 min. Half of cell samples (HT) were collected in RLT lysis buffer, while the remaining was kept overnight at 37°C with fresh culture media, generating a recovery group (HT+R). Both samples were assayed for hTGFβ and oxidative stress marker expression.

***7.5 Metabolic endoplasmic reticulum (ER) stress induction / unfolded protein response (UPR)***

The method followed the reported protocols.^13,14^ Stromal cells at about 70% confluency were treated with 2.5 μg/mL tunicamycin for 5h. Cells were collected for total RNA extraction and gene expression analysis of unfolded protein response (UPR), ER stress, apoptosis markers, and TGFβ isoforms. XBP1 splicing assay was performed by PCR followed by 2% agarose gel electrophoresis.

***8. Exosomes with proteinase K treatment***

hCSSC-Exo samples were incubated in PBS with or without 0.1% Triton X-100 and Proteinase K (50 μg/mL, Roche) for 5h at room temperature.^15^ The mixture was then added with 10 mL PBS added with 50 mM trehalose (PBS+Th) to neutralize the enzymatic activity. After spinning at 10,000g for 10 min, the supernatant was collected and ultracentrifuged at 100,000g for 2h at 4°C. Exo-enriched pellet was resuspended in PBS+Th overnight. After another centrifugation at 100,000g for 2h, the final Exo fraction was resuspended in sterile PBS+Th.

***9. hTGFB3 knockdown***

h*TGFB3* siRNA [h*TGFB3*(si)] Oligo Duplex was procured from OriGene (SR322020 with Locus ID 7043).^16^ A mixture (20 µM) of three unique 27mer siRNA duplexes for h*TGFB3* with 2 nmol each prepared in siRNA duplex resuspension buffer. One day before transfection, primary hCSSCs (HC436 and HC538) were plated in antibiotic-free growth medium to reach 30-50% confluence. RNAi duplex was diluted in Opti-MEM to 60 nM, and Lipofectamine™ RNAiMAX (Thermo Fisher) was diluted 1:50 in Opti-MEM. The mixture was incubated for 15 min at room temperature, before adding to cells. After 48 hours, cells were washed and replenished with fresh JM-H medium. Cells were collected for assessing h*TGFB* expression using qPCR and western blotting, and results were compared to cells transfected with scrambled sequence or treated with lipofectamine only. Exosomes were also purified from CM collected from h*TGFB3*(si)-transfected CSSC cultures.

***10. In vitro cell characterization assays***

***10.1 xCelligence***

The effect of TGFβ on the cell growth and shape changes was evaluated using xCelligence real-time cell analyzer RTCA SP (Agilent/ACEA, Santa Clara, CA, USA). After seeding cells at a density of 2,000 cells per well of an E-plate 96 for overnight, TGFβ1 and β3 were added at a concentration of 10 ng/mL in the respective culture medium. Medium-only cultures served as the control. The experiment was conducted in triplicate, and the impedance changes in each well was automatically recorded every 15 min for a period of 42 hours. Data were represented as cell indices for evaluation.

***10.2 In vitro fibrosis assay***

hCSKs and hSFs grown on Col1-coated coverslips were treated with CSSC-Exo at a concentration of 100 μg protein/mL for 24h prior to TGFβ1 (10 ng/mL). Controls were naïve hCSKs without TGFβ1 and Exo treatment. After 3 days, cells were fixed with ice-cold absolute ethanol for 20 min, washed, permeabilized and blocked. Samples were incubated with mouse monoclonal antibody against human αSMA (clone 1A4) (Supplementary Table S3) for 2h at room temperature. After PBS washes, the samples were incubated with AlexaFluo594-conjugated mouse IgG antibody and DAPI, mounted, and examined under confocal microscopy.

***10.3 In vitro scratch wound assay***

hSFs were seeded at a density of 2x10^4^ cells/ml in each well of a 24-well plate and cultured until near confluence. A linear wound was created by mechanically scratching the monolayer with a sterile P200 pipette tip, followed by gentle washes with plain culture medium to remove detached cells. Fresh culture media containing exosomes or mitomycin C (MMC, Sigma-Aldrich), were added to the designated wells. Experimental groups were as follows: (1) control with 5% FBS; (2) control with 5% exosome-free FBS (supernatant collected after ultracentrifugation at 100,000g for 3h at 4°C); (3) control with 0.5% exosome-free FBS; (4) unstimulated CSSC-Exo (10^7^ particles/mL); (5) M1-stimulated CSSC-Exo (10^7^ particles/mL); and (6) MMC (20 μg/mL). Both Exo and MMC were suspended in culture medium containing 0.5% exosome-free FBS. Phase-contrast images of the wound area were taken immediately (time 0) and after 12h of incubation. Cell migration was evaluated by measuring the wound width at multiple points along the scratch. The percentage of wound closure was calculated as (Wi – Wf)/Wi x100%, where Wi is the width at time 0 and Wf is the width at 12h.

***11. Exosome treatment to a mouse model of anterior stromal injury***

The research strictly adhered to guidelines outlined in the Care and Use of Laboratory Animals of the National Institutes of Health (NIH) and the Association for Research in Vision and Ophthalmology (ARVO) Statement for the Use of Animals in Ophthalmic and Vision Research. The detailed protocol was approved by the Institutional Animal Care and Use Committee (IACUC), University of Pittsburgh under Protocol 24115664 (approved on Nov 21, 2024). A total of 72 BALB/c mice (breeders from Charles River Lab., Wayne, PA) aged 10 to 12 weeks old were used. All mice were housed in AAALAC-accredited animal facility with 12h light – 12h dark cycles and provided with unrestricted food and water. They were anesthetized by intraperitoneal injection of ketamine (50 mg/kg) and xylazine (5 mg/kg) (Dechra, Fort Worth, TX).^3^ The right eyes received topical proparacaine hydrochloride (0.5%, Alcon, Fort Worth, TX) for local analgesia.

***11.1 Anterior stromal injury and exosome treatment***

After saline rinses, central corneal epithelium (2 mm diameter) was removed by a high-speed Algerbrush II (Accutome Inc, Malvern, PA). The basement membrane and anterior stroma of 0.5 mm diameter and 10 to 20 μm depth was ablated by a second Algerbrush burr (Supplementary Fig. S3).^17^ After rinsing with normal and brief drying with a sterile cotton spear, the mice were randomly assigned to treatment or control groups. The wound surface was overlaid with 1 μL fibrinogen (37 μg/mL, Sigma-Aldrich) containing CSSCs (5x10^4^ cells) and CSSC-Exo (0.5 μg protein or 10^7^ particles), respectively, followed by gelation with 0.5 μL thrombin (100 U/mL, Sigma-Aldrich). Controls were injured corneas receiving fibrin gel only or left untreated. All eyes were instilled with TobraDex eyedrops (Alcon) 3 times daily. Each group had a minimum of 6 corneas according to the sample size calculation in section 2.13.

***11.2 Ophthalmic examination and scar measurement***

Before and after injury, and at weekly intervals after treatments, mouse corneas were examined for cross-sectional changes using a Spectral Domain Optical Coherence Tomography (SD-OCT, Envisu R2210, Leica [Bioptigen], Morrisville, NC) using linear volume scan (4x4 mm area, 1024x1000x100 volumes).^18^ The scanning was performed in a masked fashion. Images were processed using NIS Elements software (Nikon). Central corneal thickness (CCT) was measured as the average of five measurements taken at the center (0 mm) and 0.5 mm on both vertical and horizontal meridians.^19^ At day 14, the mice were euthanized by overdosed intraperitoneal sodium pentobarbital followed by cervical dislocation. Cornea were isolated and imaged, and the entire corneal and scar areas were measured using ImageJ software (NIH) and MetaMorph 7.7.3 (Molecular Devices Inc.) in a blinded manner.

***12. Gene and protein expression assays***

***12.1 Immunofluorescence***

Cultured cells were fixed with freshly prepared neutral-buffered 4% paraformaldehyde for two hours or ice-cold absolute ethanol for 20 min at 4°C. After washes with 0.1M PBS, the paraformaldehyde-fixed samples were treated with ice-cold 50 mM NH_4_Cl in PBS for 10 min on ice to quench residual aldehydes. No NH_4_Cl step was applied to ethanol-fixed samples. After washes, samples were immersed in PBS containing 0.15% saponin (Sigma), 1% bovine serum albumin (BSA, Sigma), and 1.5% normal goat serum (Gibco) for 1h at room temperature to block non-specificities and to achieve permeabilization. Without washes, the samples were incubated with primary antibodies (Supplementary Table S3) for 2h at room temperature or overnight at 4°C. Following PBS washes, the staining signals were revealed by incubation with species-specific fluorescein-conjugated Ig secondary antibody and DAPI (4’,6-diamidino-2-phenylindole, 200 nM) for 1h. After PBS washes, samples were mounted with immunmount and examined under confocal microscopy (Fluoview 1200, Olympus, Center Valley, PA).

***12.2 RNA expression by qPCR***

Cells were collected in RLT buffer (Qiagen, Germantown, MD) freshly added with 1% β-mercaptoethanol, and total RNA was extracted using RNeasy Miniprep kit (Qiagen) with on-column RNase-free DNase digestion (Qiagen) per manufacturer’s instructions. For total RNA extraction from Exo samples, RLT lysate was precipitated with absolute ethanol (3.5 times of sample volume) before applying to the RNeasy spin column and all subsequent steps remained unchanged. After RNA quantification using a Nanodrop spectrophotometer (Thermo Fisher), total RNA (500 ng) was reverse transcribed with SuperScript III Reverse Transcriptase kit (Thermo Fisher) and random primer hexanucleotides (10 ng/mL). cDNA was assayed for target gene expression using SYBR Green Real-Time Master Mix (Life Technologies, Carlsbad, CA) and specific primers (Supplementary Table S2) in a QuantStudio3 Real-Time PCR System (Applied Biosyst). Experiments were performed in triplicate. Relative RNA abundance was determined using ΔCT method after normalization with housekeeping 18S (for cellular RNA) or U6 (for Exo RNA). A similar protocol was employed for extracting total RNA from mouse corneal samples, with an additional step of mechanical disruption using MagNA Lyser beads in a MagNA Lyser Instrument (Roche). The expression fold change was calculated using the 2^-ΔΔCt^ method and expressed as mean ± SD. Statistical significance was determined using a non-parametric one-way ANOVA analysis.

***12.3 Protein expression by western blotting***

Cell samples were collected in RIPA (radio-immunoprecipitation assay) buffer added with protease inhibitor cocktail (Complete^TM^, Roche) and phenylmethyl sulfonylfluoride (PMSF, 100 μM; Sigma-Aldrich). After sonication and high-speed centrifugation, supernatant was collected for total protein assay using a BCA (bicinchoninic acid) kit (Thermo Fisher). Protein samples were prepared in non-reducing Laemmli sample buffer (Bio-Rad, Hercules, CA) and resolved in a 4 to 20% gradient mini-PROTEAN TGX gel (Bio-Rad) at 120V. After transblotting on nitrocellulose membrane (0.2 µm, Bio-Rad), the blots were blocked with Licor Blocking buffer (LICORBio., Lincoln, NE), incubated with anti-human TGFβ3 and anti-human glyderaldehyde-3-phosphate dehydrogenase (GAPDH) (Supplementary Table S3), respectively, for overnight at 4°C. After washes with Tris-buffered saline containing 0.05% Tween 20 (Sigma) (TBST), the staining signals were detected with species-specific secondary anti-Ig antibodies (LICORBio.) for 1h at room temperature and revealed using a ChemiDoc MP (Thermo Fisher).

***13. miR expression in exosomes by qPCR***

Purified Exo from HC436 transfected for h*TGFB3* knockdown using siRNAs (section 9 in method), h*TGFB3* over-expression by transfection with pLVE-h*TGFB3* (Addgene),^16^ and non-transfected control were lyzed with RLT buffer and total RNA, including small RNAs, were extracted using miRNeasy kit (Qiagen) following manufacturer’s instructions. The RNA concentration and quality were measured with a NanoDrop One (Thermo Fisher Sci). cDNA was synthesized with 10 ng RNA using TaqMan MicroRNA Reverse Transcription kit (Thermo Fisher Sci). The reverse transcription program consisted of 16°C for 30 min, 45°C for 30 min, 85°C for 5 min and maintenance at 4°C. QPCR for target miRNA (has-miR-21, 29a, 155, and 381) was performed using Taqman MicroRNA Assays (**Supplementary Table S6**), following manufacturer’s instructions.^9^ No template controls were included to monitor potential contaminations. Amplifications were performed in a QuantStudio 3 QS3 PCR system under the program: 50°C for 2 min, 95°C for 10 min, followed by 40 cycles at 95°C for 15 sec and 65°C for 1 min. All reactions were conducted in triplicate. For normalization, the endogenous housekeeping hsa-miR-16 was used. Relative expression was calculated using the 2^-ΔΔCt^ method. Fold changes of target miRNAs in hCSSC-Exo were compared with that of hSF-Exo and expressed as mean ± SD. Statistical significance was determined using a non-parametric Mann-Whitney U test.

***14. Statistics***

All experiments were done in triplicate based on power analysis and experience. In mouse experiments, a minimum of 6 mice per group were investigated. The sample size was calculated using G*Power 3.1 based on the formula n=1+2C(s/d)^2^,^20^ where s is the standard deviation (SD), d is the difference to be detected, and C is the constant dependent on the value of α as the error probability 0.05 and Power (1-β err prob) 0.95. Data were presented as mean ± SD. The data normality was assessed using the Shapiro–Wilk test. For comparisons between two independent groups, an unpaired two-tailed Student’s t-test was used for normally distributed data, whereas the Mann–Whitney U test was applied for non-normally distributed (non-parametric) data. For comparisons involving more than two groups, one-way ANOVA with Bonferroni’s multiple-comparison correction was used for normally distributed data, while Kruskal–Wallis test (non-parametric alternative to one-way ANOVA) followed by Bonferroni’s correction was used for non-normally distributed data. Welch’s t-test was applied when unequal variances between groups were detected. Paired t-test was not applied as all comparisons involved independent experimental groups rather than matched samples or repeated measurements. All calculations were conducted using GraphPad Prism 11.0.2. *P*<0.05 was considered statistically significant.

**References**

1	Du, Y., Funderburgh, M. L., Mann, M. M., SundarRaj, N. & Funderburgh, J. L. Multipotent stem cells in human corneal stroma. *Stem Cells* **23**, 1266-1275 (2005).

2	Basu, S. *et al.* Human limbal biopsy-derived stromal stem cells prevent corneal scarring. *Sci Trans Med* **6**, 266ra172 (2014).

3	Santra, M. *et al.* Good manufacturing practice production of human corneal limbus-derived stromal stem cells and in vitro quality screening for therapeutic inhibition of corneal scarring. *Stem Cell Res Ther* **15**, 11 (2024).

4	Yam, G. H. *et al.* Ex vivo propagation of human corneal stromal "activated keratocytes" for tissue engineering. *Cell Transplantation* **24**, 1845-1861 (2015).

5	Riau, A. K. *et al.* Impact of keratocyte differentiation on corneal opacity resolution and visual function recovery in male rats. *Nat Comm* **15**, 4959 (2024).

6	Witwer, K. W. *et al.* Updating MISEV: Evolving the minimal requirements for studies of extracellular vesicles. *J Extracell Ves* **10**, e12182 (2021).

7	Notarangelo, M. *et al.* Ultrasensitive detection of cancer biomarkers by nickel-based isolation of polydisperse extracellular vesicles from blood. *EBioMedicine* **43**, 114-126 (2019).

8	Shojaati, G. *et al.* Mesenchymal Stem Cells Reduce Corneal Fibrosis and Inflammation via Extracellular Vesicle-Mediated Delivery of miRNA. *Stem Cells Trans Med* **8**, 1192-1201 (2019).

9	Yam, G. H. *et al.* Human corneal stromal stem cells express anti-fibrotic microRNA-29a and 381-5p - A robust cell selection tool for stem cell therapy of corneal scarring. *J Adv Res* **45**, 141-155 (2023).

10	Castro, B. *et al.* Cryopreserved H(2) O(2) -preconditioned human adipose-derived stem cells exhibit fast post-thaw recovery and enhanced bioactivity against oxidative stress. *J Tiss Eng Regen Med* **13**, 328-341 (2019).

11	Shui, C. & Scutt, A. Mild heat shock induces proliferation, alkaline phosphatase activity, and mineralization in human bone marrow stromal cells and Mg-63 cells in vitro. *J Bone Miner Res* **16**, 731-741 (2001).

12	Alekseenko, L. L. *et al.* Quiescent Human Mesenchymal Stem Cells Are More Resistant to Heat Stress than Cycling Cells. *Stem Cells Int* **2018**, 3753547 (2018).

13	Oslowski, C. M. & Urano, F. Measuring ER stress and the unfolded protein response using mammalian tissue culture system. *Methods Enzymol* **490**, 71-92 (2011).

14	Wang, X. *et al.* Induction of Fibroblast Senescence During Mouse Corneal Wound Healing. *Invest Ophthalmol Vis Sci* **60**, 3669-3679 (2019).

15	Bonsergent, E. *et al.* Quantitative characterization of extracellular vesicle uptake and content delivery within mammalian cells. *Nat Comm* **12**, 1864 (2021).

16	Weng, L. *et al.* The anti-scarring effect of corneal stromal stem cell therapy is mediated by transforming growth factor β3. *Eye Vis (Lond)* **7**, 52 (2020).

17	Khandaker, I. *et al.* A novel transgenic mouse model for corneal scar visualization. *Exp Eye Res* **200**, 108270 (2020).

18	Lathrop, K. L. *et al.* Human Corneal Stromal Stem Cell Treatment Reduces Established Opacities in Chronic Corneal Scarring. *Cells* **15** (2026).

19	Yam, G. H. *et al.* Safety and Feasibility of Intrastromal Injection of Cultivated Human Corneal Stromal Keratocytes as Cell-Based Therapy for Corneal Opacities. *Invest Ophthalmol Vis Sci* **59**, 3340-3354 (2018).

20	Faul, F., Erdfelder, E., Buchner, A. & Lang, A. G. Statistical power analyses using G*Power 3.1: tests for correlation and regression analyses. *Behav Res Methods* **41**, 1149-1160 (2009).

21	Arrick, B. A., Lee, A. L., Grendell, R. L. & Derynck, R. Inhibition of translation of transforming growth factor-β3 mRNA by its 5' untranslated region. *Mol Cell Biol* **11**, 4306-4313 (1991).

**Supplementary Figures**

**Supplementary Figure S1. Schematic representation of human transforming growth factor β3 (TGFβ3) peptide NP_001316868.1.** The full-length peptide with latent region is indicated by red asterisks.

**Human TGFB3 mRNA** NM_001329939.2-gene

Up/downstream 14:75,958,097-75,983,011(-) +/- 500 up/downstream bp

Up/Downstream

UTR

Coding

Amino acids

gtgcctggtatatagtaaacattaaactttttctgaaatttcagaggcagtatagcatagtaattaagtccagaatctggcaacgtcctgggtccaaatcccaacagctgacacctaataactatgtgaccttgggcaagttacttttaaagtttctacccctaggtttcccattggttttgcaaatgaaagtaatgcctacccaagctagatagcctgtgtaaaatatcgcctccatcactcacaagcagtgtggtctgtaaaaaaaaaaacaaaaaactctatgcctcagtttcctcatccgtaaagtgacccaccgctgtgctgggatacagagaacagccccttcagttagtggcctggaagccagacctctcagaaagggtccaggaaggctggagtgagatggggtgggagcggcactcactctcaggaaagttcagttcagaggcaagccctgtgttgcggggtgcggggagccacgtgccctaccctccctt

1	ggctgctcgt ggg**aaaaggc ctagaggttc ggg**ccgagaa gaggagcgaa agcacagagc cgacttcccc tcacccatct gggaaatggg ctcgggccaa ctgctgactt cgcgctcgct

121	ggcgcagctc cctgcggaga cctcggcggg gagggaggct gaacatctgg atgacatttc tgcgagagcg gctccggagc ggcggtcggg gagggagag

gtgcgctcgtgcgcacgtcgggccgggagggaggcgattcctcggggcctgggtcttgtttttctcgctctctaccgcagccccttctcccgcccctcagcccccaccccgcagcccccagcccccgagcctccccggctcccgaccagccgagctccttcactggcggcctcgcctcgccagagggcaccctcgatcttccggaaaacgccaccatttttcactgcccctggagcgtctccaggcttctgcccgcctcccgactccgatcttgtcaatgaagaatcgggccaggatcgccgcggagcggacgccgaccctccgacccggctcgcaggctgggagtcccctctgcgaggctggcatggccgcccctaccgggtcccgcgccctctgcggaccctgccccgggttgggcctggcccgcgggcggccccgggaccgggggaccaggagggagagtagacgcgggccgcggacggcgcggactgacagctggcgagagggcgccggggctgggggaaagggagggagggggctcatcggagtaactttccagaaaaacagccaacgtgtggcaggagtgattccaagaggggaaaaaaagttcagctaccacgtcgaacgagaggactcgcaaagtatttttcaaaagggctcggcttttcctgtgcctgtttaaaacattaacatcgtgcagcaaaagaggctgcgtgcgctggtccctccctcccccaccccaggccagagacgtcatgggagggagg**tataaaa**tttcagcagagagaaatagagaaagcagtgtgtgtgcatgtgtgtgtgtgtgagagagagagggagaggagcgagagggagagggag	**----------->**

agggagagagagaaagggagggaagcagagagtcaagtccaagggaatgagcgagagaggcagagacaggggaagaggcgtgcgagagaaggaataacagctttccggagcaggcgtgccgtgaactggcttctattttattttatttttttctcctttttattttttaaaga gaagcagggg acagaagcaa

241	tggccgaggc agaagacaag ccgaggtgct ggtgaccctg ggcgtctgag tggatgattg gggctgctgc gctcagaggc ctgcctccct gccttccaat gcatataacc ccacac**ccca**

361	**gccaatgaag acgaga**ggca gcgtgaacaa agtcatttag aaagcccccg aggaagtgta aacaaaagag aaagcatgaa tggagtgcct gagagacaag tgtgtcctgt actgccccca

481	cctttagctg ggccagcaac tgcccggccc tgcttctccc cacctactca ctggtgatct tttttttttt actttttttt cccttttctt ttccattctc ttttcttatt ttctttcaag

601	gcaaggcaag gattttgatt ttgggaccca gccatggtcc ttctgcttct tctttaaaat acccactttc tccccatcgc caagcggcgt ttggcaatat cagatatcca ctctatttat

721	ttttacctaa ggaaaaactc cagctccctt cccactccca gctgccttgc cacccctccc agccctctgc ttgccctcca cctggcctgc tgggagtcag agcccagcaa aacctgttta

841	gacacatgga caagaatccc agcgctacaa ggcacacagt ccgcttcttc gtcctcaggg ttgccagcgc ttcctggaag tcctgaagct ctcgcagtgc agtgagttca tgcaccttct

961	tgccaagcct cagtctttgg gatctgggga ggccgcctgg ttttcctccc tccttctgca cgtctgctgg ggtctcttcc tctccaggcc ttgccgtccc cctggcctct cttcccagct

1081	cacac **ATG AAG ATG CAC TTG CAA AGG GC**T CTG GTG GTC CTG GCC CTG CTG AAC TTT GCC A

Met Lys	Met His	Leu Gln Arg Ala	Leu Val	Val	Leu Ala Leu Leu Asn Phe Ala

1141	CG GTC AGC CTC TCT CTG TCC ACT TGC ACC ACC TTG GAC TTC GGC CAC ATC AAG AAG AAG A

1201	GG GTG GAA GCC ATT AGG GGA CAG ATC TTG AGC AAG CTC AGG CTC ACC AGC CCC CCT GAG C

1261	CA ACG GTG ATG ACC CAC GTC CCC TAT CAG GTC CTG GCC CTT TAC AAC AGC ACC CGG GAG C

1321	TG CTG GAG GAG ATG CAT GGG GAG AGG GAG GAA GGC TGC ACC CAG GAA AAC ACC GAG TCG G

1381	AA TAC TAT GCC AAA GAA ATC CAT AAA TTC GAC ATG ATC CAG GGG CTG GCG GAG CAC AAC G

1441	AA CTG GCT GTC TGC CCT AAA GGA ATT ACC TCC AAG GTT TTC CGC TTC AAT GTG TCC TCA G

1501	TG GAG AAA AAT AGA ACC AAC CTA TTC CGA GCA GAA TTC CGG GTC TTG CGG GTG CCC AAC C

1561	CC AGC TCT AAG CGG AAT GAG CAG AGG ATC GAG CTC TTC CAG ATC CTT CGG CCA GAT GAG C

1621	AC ATT GCC AAA CAG CGC TAT ATC GGT GGC AAG AAT CTG CCC ACA CGG GGC ACT GCC GAG T

1681	GG CTG TCC TTT GAT GTC ACT GAC ACT GTG CGT GAG TGG CTG TTG AGA AGA GAG TCC AAC T

1741	TA GGT CTA GAA ATC AGC ATT CAC TGT CCA TGT CAC ACC TTT CAG CCC AAT GGA GAT ATC C

1801	TG GAA AAC ATT CAC GAG GTG ATG GAA ATC AAA TTC AAA GGC GTG GAC AAT GAG GAT GAC C

1861	AT GGC CGT GGA GAT CTG GGG CGC CTC AAG AAG CAG AAG GAT CAC CAC AAC CCT CAT CTA A

1921	TC CTC ATG ATG ATT CCC CCA CAC CGG CTC GAC AAC CCG GGC CAG GGG GGT CAG AGG AAG A

1981	AG CGG GCT TTG GAC ACC AAT TAC TGC TTC CGC AAC TTG GAG GAG AAC TGC TGT GTG CGC C

2041	CC CTC TAC ATT GAC TTC CGA CAG GAT CTG GGC TGG AAG TGG GTC CAT GAA CCT AAG GGC T

2101	AC TAT GCC AAC TTC TGC TCA GGC CCT TGC CCA TAC CTC CGC AGT GCA GAC ACA ACC CAC A

2161	GC ACG GTG CTG GGA CTG TAC AAC ACT CTG AAC CCT GAA GCA TCT GCC TCG CCT TGC TGC G

2221	TG CCC CAG GAC CTG GAG CCC CTG ACC ATC CTG TAC TAT GTT GGG AGG ACC CCC AAA GTG G

2281	AG CAG CTC TCC AAC ATG **GTG GTG AAG TCT TGT AAA TGT AGC TGA** gacccc acgtgcgaca

2341	gagagagggg agagagaacc accactgcct gactgcccgc tcctcgggaa acacacaagc aacaaacctc actgagaggc ctggagccca caaccttcgg ctccgggcaa atggctgaga

2461	tggaggtttc cttttggaac atttctttct tgctggctct gagaatcacg gtggtaaaga aagtgtgggt ttggttagag gaaggctgaa ctcttcagaa cacacagact ttctgtgacg

2581	cagacagagg ggatggggat agaggaaagg gatggtaagt tgagatgttg tgtggcaatg ggatttgggc taccctaaag ggagaaggaa gggcagagaa tggctgggtc agggccagac

2701	tggaagacac ttcagatctg aggttggatt tgctcattgc tgtaccacat ctgctctagg gaatctggat tatgttatac aaggcaagca tttttttttt ttttttaaag acaggttacg

2821	aagacaaagt cccagaattg tatctcatac tgtctgggat taagggcaaa tctattactt ttgcaaactg tcctctacat caattaacat cgtgggtcac tacagggaga aaatccaggt

2941	catgcagttc ctggcccatc aactgtattg ggccttttgg atatgctgaa cgcagaagaa agggtggaaa tcaaccctct cctgtctgcc ctctgggtcc ctcctctcac ctctccctcg

3061	atcatatttc ccct**tggaca cttggttaga cgcc**ttccag gtcaggatgc acatttctgg attgtggttc catgcagcct tggggcatta tgggttcttc ccccacttcc **cctccaagac**

3181	**cctgtgttc**a tttggtgttc ctggaagcag gtgctacaac atgtgaggca ttcggggaag ctgcacatgt gccacacagt gacttggccc cagacgcata gactgaggta taaagacaag

3301	tatgaatatt actctcaaaa tctttgtata aataaatatt tttggggcat cctggatgat ttcatcttct ggaatattgtt tctagaacag taaaagcctt attctaaggt gta

tgtctgactcgataaatatccttcaattacccttaattccatgtccgcatctactaatcagaggtaaccagaatctggggcagagaatctgtcaatcaccaaacacattgcttagcgcaactccccttacacagggggcacaccgtgctgacttccgcctgctaagaagactgcactattgcttgtgcgtctttccttcttgcagagtatattattctcagagacacagaggcagtggctcagattggcagaaagcacatacgaatttgacctccagatacctgtgggcaggatcccctggtgtgaatccttgcatatggaaacatggtttatttactaactataaattaccaacatcactgttttcgaaaatctccccccacccgtatcagttgagaatgagagaaaatgtgtatggcaaatggcctaaaaatatgaggctaattctgttctcaggctaagcctaaaagagctaaccaggaaccccttttcagattgtg

**Supplementary Figure S2. Nucleotide sequence of human *TGFB3* mRNA and franking sequences, including 5’ and 3’ untranslated region (UTR) (NM_001329939.2).** The TATA box is underlined and the transcription start site is indicated by a dotted-line arrow**.**^21^ Blue-marked regions indicate 5’UTR before the start codon ATG (bold and enlarged size, nucleotide +1085) and 3’UTR following the stop codon TGA (bold and enlarged font size, nucleotide +2324), respectively. Protein coding regions are marked in yellow (first 20 amino acids are indicated) and nucleotides are capitalized. PCR primers specific to 5’ and 3’UTR, and full-length protein-coding sequence and the locations are listed.

hTGFB3_protein-coding-F	5’-**ATGAAGATGCACTTGCAAAGGGC**

hTGFB3_protein-coding-R	5’-**TCAGCTACATTTACAAGACTTCACCAC**

Amplicon: 1239 bp (nt1086-2324)

hTGFB3_5UTR-F	5’-**AAAAGGCCTAGAGGTTCGGG**

hTGFB3_5UTR-R	5’-**TCTCGTCTTCATTGGCTGGG**

Amplicon: 363 bp (nt14-376)

hTGFB3_3UTR (with stop)-F	5’-GAGCCCCTGACCATCCTGTA

hTGFB3_3UTR (with stop)-R	5’-CGTGGGGTCTCAGCTACATT

Amplicon: 99 bp (nt2235-2333)

hTGFB3_3UTR-F	5’-**TGGACACTTGGTTAGACGCC**

hTGFB3_3UTR-R	5’-**GAACACAGGGTCTTGGAGGG**

Amplicon: 115 bp (nt3075-3189)

**Supplementary Figure S3. A mouse model of corneal anterior stromal injury and CSSC treatment.** Using an Algerbrush burr to create an epithelial wound, the injury disrupted the Bowman's membrane and produced anterior stromal wound of 0.5 mm diameter and 20-30 μm depth. Immediately after injury and washes, cells or exosomes were applied in a fibrin gel (fibrinogen and thrombin). OCT images at the bottom panel show the cross-sectional cornea before and after injury as well as post-treatment with the exosome/fibrin drop on the wound surface.

**Supplementary Figure S4. M1 and M2 polarization of mouse macrophage RAW264.7 cells.** Treatment with lipopolysaccharides (LPS, 25 ng/mL) to induce pro-inflammatory M1 phenotype (*INOS* and *MCP1* upregulation). Treatment with interleukin-4 (IL4, 20 ng/mL) to induce M2 phenotype (*ARG1* and *PTGS1* upregulation). Results were compared with non-treated cells (M0). After M1 and M2 induction and PBS washes, the cultures were replenished with plain medium for 24h and conditioned media were collected for concentration using MicroCon centrifugal filters (10k cut-off range) down to 1/20^th^ original volumes.

**Supplementary Figure S5. *In vitro* non-lethal stress induction.** Donor CSSCs were treated with different non-lethal cell stressors. (1) Serum-free or nutrient deprivation for 48h; (2) M1 pro-inflammatory stimulation for 48h; (3) transient heat-shock at 40°C for 90 min and recovered to 37°C; (4) oxidative insult with 20 μM hydrogen peroxide (H_2_O_2_) for 6 days; and (5) metabolic endoplasmic reticulum (ER) stress with tunicamycin (2.5 μg/mL) for 5h. Cells were harvested to assess the expression changes of *TGFB3* compared with non-treated CSSCs. Our results showed that *TGFB3* was upregulated after nutrient deprivation and M1 pro-inflammatory stimulation, whereas no changes were found in samples with transient heat-shock, oxidative insult, and metabolic ER stress.

**Supplementary Figure S6**. XBP1 splicing assay measures the unconventional splicing of *XBP1* mRNA, a key event in the unfolded protein response to endoplasmic reticulum stress. After PCR amplification, both non-spliced (442 base pairs; *uXBP1*) and spliced *XBP1* amplicons (416 base pairs; *sXBP1*) was detected in cell samples after tunicamycin (TM) treatment while non-spliced *XBP1* only in the untreated samples. Band densitometry showed the significant occurrence of *sXBP1* in TM-treated cells, with an average of 55±14% spliced form intensity, compared with untreated controls (*P*<0.001, Mann-Whitney U test).

**Supplementary Figure S7**. Unfolded protein response and ER stress marker expression in donor CSSCs after treatment of transient stressors – metabolic stress by tunicamycin treatment, oxidative stress by H_2_O_2_ treatment, and transient hyperthermia with or without recovery. The expression of genes involved in protein folding (*BIP/GRP78*), apoptosis (*CHOP* and *PERK*), and ER stress regulation (*ATF4* and *ATF6*) were upregulated in cells after tunicamycin treatment. No changes were observed for the treatment with other stressors.

**Supplementary Figure S8**. **Human CSSC-derived exosome characterization.** (**A**) Representative transmission electron micrograph showing spherical morphology of exosomes. Scale bar: 50 nm. (**B**) Size histogram measured by the TRPS assay. The size range was less than 200 nm with peak size around 67-88 nm.

**Supplementary Figure S9**. **Flow cytometry and western blot analyses of membrane marker expression in donor CSSC-derived exosomes.** (**A**) Distinct peaks were noted for exosome marker, CD81. (**B**) Distinct bands of CD9, CD63, and CD81 were detected in exosome fractions prepared from different donor CSSC batches.

**Supplementary Figure S10**. **Uptake of exosomes by stromal keratocytes.** CFSE-labeled CSSC-Exo were captured by CSKs and assumed a cytoplasmic localization. Scale bar: 10 μm.

**Supplementary Figure S11**. **Treatment with hCSSC-Exo with or without Proteinase K pre-treatment suppressed *in vitro* fibrosis.** hCSKs treated with recombinant human TGFβ1 (10 ng/mL) for 3 days exhibited myoF formation, indicated by increased αSMA expression. Cells pre-treated with hCSSC-Exo with or without Proteinase K (50 μg/mL for 5h) exhibited comparable downregulation of αSMA expression induced by TGFβ1. Quantification analysis of αSMA positive cells in five random fields (each 400x400 μm^2^) showed significant suppression of αSMA expression in hCSKs pre-incubated with CSSC-Exo treated or not with Proteinase K, alongside TGFβ1, compared to TGFβ1 alone (**P*<0.05; Mann-Whitney U test). Scale bar: 25 μm.

**Supplementary Figure S12. M1-CSSC-Exo suppressed hSF growth and migration**. (**A**) xCelligence analysis shows the cell index profiles of SF under various treatments. 1 - control with 5% FBS; 2 - control with 5% Exo-free FBS; 3 - control with 0.5% exosome-free FBS; 4 - unstimulated CSSC-Exo (10^7^ particles/mL); 5 - M1-stimulated CSSC-Exo (10^7^ particles/mL); 6 – mitomycin C (MMC, 2 μg/mL). Both Exo and MMC were suspended in culture medium containing 0.5% Exo-free FBS. (**B**) Treatment with M1-stimulated CSSC-Exo, unstimulated CSSC-Exo, and MMC significantly increased the cell doubling time, as calculated from cell indices. (**C**) An *in vitro* scratch-wound assay on SF culture. Representative phase-contrast images at time 0 and at 12h following treatment with M1-stimulated and unstimulated CSSC-Exo, MMC, as well as controls with Exo-free FBS alone (** *P*<0.01; Mann-Whitney U test) (**D**) Percentages of wound width at 12h after Exo and MMC treatments are presented. Cultures treated with Exo showed apparent wound widths similar as those with low-dose MMC treatment (10 ng/mL), both significantly greater than control, indicating Exo and MMC treatments delayed wound closure (* *P*<0.05; Kruskal–Wallis test).

**Supplementary Figure S13. Treatment with M1-stimulated CSSC-Exo.** Mouse corneas collected at day 10 post-injury, and cell treatment with different donor CSSC (5x10^4^ cells; HC436, HC439, HC641) or their M1-stimulated CSSC-Exo (10^7^ particles) in fibrin gel. Heat-denatured Exo from HC439 lost the scar-inhibitory effect.

**Supplementary Figure S14. Treatment with hCSSC-Exo with or without Proteinase K pre-treatment inhibited corneal scarring.** Mouse corneas collected at day 10 post-injury, followed by treatment with CSSC-Exo (10^7^ particles) in fibrin gel. Exo pre-treated or not with Proteinase K markedly reduced opacity formation, compared with injury only and fibrin vehicle sham control (**P*<0.05, Mann-Whitney U test).

**Supplementary Figure S15. Original western blot images** for hTGFβ3 and housekeeping GAPDH. Primary donor CSSCs HC436 and HC439 at P3 were transfected with h*TGFB3*(si) to achieve hTGFβ3 knockdown. Controls (C) were cells transfected with scrambled sequences. hTGFβ3 proprotein was detected as around 50 kDa bands whereas GAPDH bands were at 37 kDa. All samples were loaded at 25 μg total protein. Recombinant hTGFβ3 (rhTGFβ3) served as the positive staining control and 0.5 μg protein was loaded.

**Supplementary Figure S16. Expression of exosomal microRNAs after h*TGFB3* knockdown or overexpression (O/E).** Purified Exo from hCSSCs (HC436) with h*TGFB3* knockdown by siRNA-mediated transfection showed insignificant changes in the expression of hsa-miR-21, 29a, 155, and 381 (after normalization with housekeeping miR-16) when compared with Exo from naïve parental cells. In contrast, h*TGFB3* overexpression selectively downregulated hsa-miR-21, 29a, and 381. Fold changes of microRNAs in Exo436 were calculated against that of hSF-Exo. **P*<0.05, Mann-Whitney U test.

**Supplementary Figure S17.** Full-sized confocal images showing immunofluorescence of αSMA and FN in cells treated with varying TGFβ3/β1 ratios for 48h. The numbers in brackets represent growth factor dosage at ng/mL. Scale bar: 150 μm.

**Supplementary Table S1. Donor cornea information**

| Lab no. | Age | Gender | Cause of Death | Days in Optisol preservation |
| --- | --- | --- | --- | --- |
| 436 | 33 | M | Multiple cerebral infarcts | 7 |
| 439 | 57 | F | Anoxia brain injury | 9 |
| 461 | 18 | M | Gun shot | 8 |
| 462 | 66 | M | Myocadiac infarct | 7 |
| 466 | 51 | F | Myocardial infarct | 8 |
| 515 | 67 | M | Atherosclerotic cardiovascular disease | 6 |
| 534 | 25 | M | Head trauma | 9 |
| 538 | 62 | F | Decompensated heart failure | 9 |
| 540 | 57 | F | STEMI heart attack | 8 |
| 572 | 21 | M | Anoxia brain injury | 10 |
| 579 | 52 | M | Motor vehicle accident | 8 |
| 584 | 55 | F | Myocadiac infarct | 8 |
| 616 | 55 | F | Multiple cerebral infarcts | 9 |
| 623 | 45 | F | Motor vehicle accident | 9 |
| 624 | 65 | M | Hypoxic ischemic encephalopathy | 10 |
| 641 | 45 | M | Gun shot | 9 |
| 647 | 51 | M | Multiple cerebral infarcts | 10 |
| 652 | 56 | F | Myocadiac infarct | 10 |
| 670 | 49 | F | Head trauma | 9 |

**Supplementary Table S2:** PCR primers

| Gene Name | **Primer Sequences** | **GenBank Accession No.** | **Amplicon size** |
| --- | --- | --- | --- |
| A. Human stem cell markers | | | |
| *ABCG2* | Forward: TGCAACATGTACTGGCGAAGA  Reverse: TCTTCCACAAGCCCCAGG | NM_001348989.2 | 71 bp |
| *NES* | Forward: AAGATGTCCCTCAGCCTGG  Reverse: GAGGGAAGTCTTGGAGCCAC | NM_006617.2 | 99 bp |
| *PAX6* | Forward: CAATCAAAACGTGTCCAACG  Reverse: TAGCCAGGTTGCGAAGAACT | [NM_001368892.2](https://www.ncbi.nlm.nih.gov/entrez/viewer.fcgi?db=nucleotide&id=1844161464) | 263 bp |
| *OCT4* | Forward: GTGGAGGAAGCTGACAACAA  Reverse: GGTTCTCGATACTGGTTCGC | NM_001173531.3 | 97 bp |
| *SOX2* | Forward: AACCCCAAGATGCACAACTC  Reverse: GCTTAGCCTCGTCGATGAAC | NM_003106.4 | 100 bp |
| B. Human TGFβ isoforms | | | |
| *TGFB1* | Forward: CCCTGGACACCAACTATTGC  Reverse: AAGTTGGCATGGTAGCCCT | NM_000660.7 | 127 bp |
| *TGFB2* | Forward: ATAGACATGCCGCCCTTCTT  Reverse: CTCCATTGCTGAGACGTCAA | NM_001135599.4 | 183 bp |
| *TGFB3* | Forward: ATGATGATTCCCCCACACCG  Reverse: TCCAAGTTGCGGAAGCAGTA | [NM_003239.5](https://www.ncbi.nlm.nih.gov/entrez/viewer.fcgi?db=nucleotide&id=1809665871) | 95 bp |
| Protein-coding *TGFB3* | Forward: ATGAAGATGCACTTGCAAAGGGC  Reverse: TCAGCTACATTTACAAGACTTCACCAC | NM_001329939.2 | 1239 bp |
| *TGFB3* 5’UTR | Forward: AAAAGGCCTAGAGGTTCGGG  Reverse: TCTCGTCTTCATTGGCTGGG | NM_001329939.2 | 363 bp |
| *TGFB3* 3’UTR | Forward: TGGACACTTGGTTAGACGCC  Reverse: GAACACAGGGTCTTGGAGGG | NM_001329939.2 | 115 bp |
| C. Human ER stress markers | | | |
| *XBP1* | Forward: CCTTGTAGTTGAGAACCAGG  Reverse: GGGGCTTGGTATATATGTGG | [NM_005080.4](https://www.ncbi.nlm.nih.gov/entrez/viewer.fcgi?db=nucleotide&id=1889619472) | 442 bp (uXBP1)  416 bp (sXBP1) |
| *BIP* | Forward: CTGTCCAGGCTGGTGTGCTCT  Reverse: CTTGGTAGGCACCACTGTGTTC | BC020235.1 | 143 bp |
| *CHOP* | Forward: GCCTTTCTCCTTTGGGACACTGTCCAG  Reverse: CTCGGCGAGTCGCCTCTACTTCCC | BT006691.1 | 422 bp |
| *PERK* | Forward: GTCCGGAACCAGACGATGAG  Reverse: GGCTGGATGACACCAAGGAA | BC126356.1 | 159 bp |
| *BAX* | Forward: TCAGGATGCGTCCACCAAGAA  Reverse: TGTGTCCACGGCGGCAATCATC | OQ348809.1 | 102 bp |
| *BCL2* | Forward: ATCGCCCTGTGGATGACTGAG  Reverse: GCCAGGAGAAATCAAACAGAG | NM_000633.1 | 121 bp |
| D. Human fibrotic and inflammatory markers | | | |
| *ACTA2* | Forward: CTGTTCCAGCCATCCTTCAT  Reverse: CCGTGATCTCCTTCTGCATT | NM_001613.2 | 175 bp |
| *COL3A1* | Forward: TCTTTGAATCCTAGCCCATCTG  Reverse: TGTGACAAAAGCAGCCCCATAA | NM_000090.3 | 569 bp |
| *FN* | Forward: CCACCCCCATAAGGCATAGG  Reverse: GTAGGGGTCAAAGCACGAGTCATC | NM_212482.4 | 178 bp |
| *MCP1* | Forward: GAGAGGCTGAGACTAACCCAGA  Reverse: ATCACAGCTTCTTTGGGACACT | NM_002982.4 | 155 bp |
| *SPARC* | Forward: ATCTAAATCCACTCCTTCCACAG  Reverse: CACCGTTAATGTATTCACTTAAAT | NM_003118.4 | 134 bp |
| E. Human keratocyte markers | | | |
| *KERA* | Forward: ATCTGCAGCACCTTCACCTT  Reverse: CATTGGAATTGGTGGTTTGA | NM_007035.3 | 167 bp |
| *LUM* | Forward: CCTGGTTGAGCTGGATCTGT  Reverse: TGGTTTCTGAGATGCGATTG | NM_002345.3 | 194 bp |

| Mouse Oligos | | | |
| --- | --- | --- | --- |
| Gene Name | **Primer Sequences** | **GenBank Accession No.** | **Amplicon size** |
| *COL3A1* | Forward: CGTAAGCACTGGTGGACAGA  Reverse: CGGCTGGAAAGAAGTCTGAG | NM_009930.2 | 155 bp |
| *FN1* | Forward: TACTCGAGCCCTGAGGATGG Reverse: GCAAGGCAACCACACTGACT | NM_010233.2 | 120 bp |
| *TNC* | Forward: GACTGCCCTGGGAACTGTAA  Reverse: CATAGCCTTCGAAGCACACA | NM_035737.2 | 162 bp |
| *iNOS* | Forward: TCACCTTCGAGGGCAGCCGA  Reverse: TCCGTGGCAAAGCGAGCCAG | NM_001313922.1 | 286 bp |
| *MCP1* | Forward: TGATCCCAATGAGTAGGCTGGAG  Reverse: ATGTCTGGACCCATTCCTTCTTG | NM_011333.3 | 132 bp |

**Supplementary Table S3:** Antibodies used in this study

| Antibody (clone) | Sources | Applications in this study |
| --- | --- | --- |
| Anti-human CD73-FITC conjugate (AD2) | BioLegend 344015 | Flow cytometry |
| Anti-human CD90-FITC conjugate (5E10) | BioLegend 328107 | Flow cytometry |
| Anti-human CD105-FITC conjugate (43A3) | BioLegend 323203 | Flow cytometry |
| Mouse anti-human CD9 (TS9) | Invitrogen 10626D | Western blotting |
| Mouse anti-human CD63 (Ts63) | Invitrogen 10628D | Western blotting |
| Mouse anti-human CD81 (1.3.3.22) | Thermo Fisher MAS-13548 | Western blotting |
| Mouse anti-human αSMA (1A4) | Sigma A2547 | Immunofluorescence |
| Goat anti-human Col3A1 (C-15) | Santa Cruz sc-8781 | Immunofluorescence |
| Rabbit anti-human keratocan (KERA) | Sigma HPA039321 | Immunofluorescence |
| Rabbit anti-human fibronectin (FN) | Abcam 2413 | Immunofluorescence |
| Rabbit anti-human lumican | Sigma HPA001522 | Immunofluorescence |
| Rabbit anti-human TGFß3 | Sigma SAB2103852 | Flow cytometry, immunofluorescence |

**Supplementary Table S4. Donor CSSCs and exosomes used in this study**

| CSSC | CSSC-derived exosomes | Uses in experiments | |
| --- | --- | --- | --- |
|  |  | ***In vitro*** | ***In vivo*** |
| HC436 | Exo436 | Yes | Yes (Exo and cells) |
| HC439 | Exo439 | Yes | Yes (Exo and cells) |
| HC461 | Exo461 | Yes | Yes (Exo) |
| HC462 | Exo462 | Yes | Yes (Exo) |
| HC466L | Exo466L | Yes | -- |
| HC515 | Exo515 | Yes | -- |
| HC534 | Exo534 | Yes | -- |
| HC538 | Exo538 | Yes | -- |
| HC540 | Exo540 | Yes | -- |
| HC572 | Exo572 | Yes | -- |
| HC623 | Exo623 | Yes | -- |
| HC624 | Exo624 | Yes | Yes (Exo) |
| HC641 | Exo641 | Yes | Yes (Exo and cells) |
| HC584 |  | Yes |  |
| HC616 |  | Yes |  |
| HC647 |  | Yes |  |

**Supplementary Table S5. Donor CSKs and SFs used in this study**

| Donor corneas | CSK batches | SF batches | Uses in experiments |
| --- | --- | --- | --- |
| C579 | CSK579 | SF579 | *In vitro* fibrosis, scratch-wound assays |
| C584 | CSK584 | SF584 | *In vitro* fibrosis assays |
| C670 | CSK670 | SF670 | *In vitro* fibrosis, scratch-wound assays |
|  | -- | YD/67 | xCelligence and scratch-wound assays |
|  | -- | SF652 | xCelligence and scratch-wound assays |

**Supplementary Table S6. MicroRNA Taqman assays**

| **Assay** | **miRbase Accession No.** | **Assay ID (Thermo Fisher Sci)** |
| --- | --- | --- |
| hsa-miR-16 | MI0000070 | 000391 |
| hsa-miR-21 | MI0000077 | 002438 |
| hsa-miR-29a | MI0000087 | 002112 |
| hsa-miR-155 | MI0000681 | 002287 |
| hsa-miR-381 | MI0000789 | 463623_mat |
